# Internet and Telephone Support for Discontinuing Long-Term Antidepressants: The REDUCE Cluster Randomized Trial

**DOI:** 10.1001/jamanetworkopen.2024.18383

**Published:** 2024-06-24

**Authors:** Tony Kendrick, Beth Stuart, Hannah Bowers, Mahboobeh Haji Sadeghi, Helen Page, Christopher Dowrick, Michael Moore, Mark Gabbay, Geraldine M. Leydon, Guiqing Lily Yao, Paul Little, Gareth Griffiths, Glyn Lewis, Carl May, Joanna Moncrieff, Chris F. Johnson, Una Macleod, Simon Gilbody, Rachel Dewar-Haggart, Samantha Williams, Wendy O’Brien, Riya Tiwari, Catherine Woods, Tasneem Patel, Naila Khan, Nadja van Ginneken, Amy Din, Claire Reidy, Rebecca Lucier, Bryan Palmer, Taeko Becque, Ellen van Leeuwen, Shihua Zhu, Adam W. A. Geraghty

**Affiliations:** 1 Primary Care Research Centre, School of Primary Care, Population Health & Medical Education, Faculty of Medicine, University of Southampton, Aldermoor Health Centre, Southampton, United Kingdom; 2 Centre for Evaluation and Methods, Wolfson Institute of Population Health, Faculty of Medicine and Dentistry, Queen Mary University of London, London, United Kingdom; 3 Institute for Clinical and Applied Health Research, Hull-York Medical School, University of Hull, Hull, United Kingdom; 4 Department of Primary Care and Mental Health, University of Liverpool, Liverpool, United Kingdom; 5 Leicester Clinical Trials Unit, University of Leicester, Leicester, United Kingdom; 6 Southampton Clinical Trials Unit, University of Southampton and University Hospital Southampton National Health Service (NHS) Foundation Trust, Southampton, United Kingdom; 7 Division of Psychiatry, University College London, London, United Kingdom; 8 Faculty of Public Health and Policy, London School of Hygiene and Tropical Medicine, London, United Kingdom; 9 Primary Care Pharmacy Services, NHS Greater Glasgow & Clyde, Glasgow, United Kingdom; 10 Mental Health & Addiction Research Group, Department of Health Sciences, University of York, York, United Kingdom; 11 Patient and Public Contributor, Southampton, United Kingdom; 12 Department of Basic and Applied Medical Sciences, Ghent University, Ghent, Belgium; 13 Department of Public Health and Primary Care, Ghent University, Ghent, Belgium; 14 Community and Health Research Unit, School of Health & Social Care, University of Lincoln, Lincoln, United Kingdom

## Abstract

**Importance:**

There is significant concern regarding increasing long-term antidepressant treatment for depression beyond an evidence-based duration.

**Objective:**

To determine whether adding internet and telephone support to a family practitioner review to consider discontinuing long-term antidepressant treatment is safe and more effective than a practitioner review alone.

**Design, Setting, and Participants:**

In this cluster randomized clinical trial, 131 UK family practices were randomized between December 1, 2018, and March 31, 2022, with remote computerized allocation and 12 months of follow-up. Participants and researchers were aware of allocation, but analysis was blind. Participants were adults who were receiving antidepressants for more than 1 year for a first episode of depression or more than 2 years for recurrent depression who were currently well enough to consider discontinuation and wished to do so and who were at low risk of relapse. Of 6725 patients mailed invitations, 330 (4.9%) were eligible and consented.

**Interventions:**

Internet and telephone self-management support, codesigned and coproduced with patients and practitioners.

**Main Outcomes and Measures:**

The primary (safety) outcome was depression at 6 months (prespecified complete-case analysis), testing for noninferiority of the intervention to under 2 points on the 9-item Patient Health Questionnaire (PHQ-9). Secondary outcomes (testing for superiority) were antidepressant discontinuation, anxiety, quality of life, antidepressant withdrawal symptoms, mental well-being, enablement, satisfaction, use of health care services, and adverse events. Analyses for the main outcomes were performed on a complete-case basis, and multiple imputation sensitivity analysis was performed on an intention-to-treat basis.

**Results:**

Of 330 participants recruited (325 eligible for inclusion; 178 in intervention practices and 147 in control practices; mean [SD] age at baseline, 54.0 [14.9] years; 223 women [68.6%]), 276 (83.6%) were followed up at 6 months, and 240 (72.7%) at 12 months. The intervention proved noninferior; mean (SD) PHQ-9 scores at 6 months were slightly lower in the intervention arm than in the control arm in the complete-case analysis (4.0 [4.3] vs 5.0 [4.7]; adjusted difference, −1.1; 95% CI, −2.1 to −0.1; *P* = .03) but not significantly different in an intention-to-treat multiple imputation sensitivity analysis (adjusted difference, −0.9 (95% CI, −1.9 to 0.1; *P* = .08). By 6 months, antidepressants had been discontinued by 66 of 145 intervention arm participants (45.5%) who provided discontinuation data and 54 of 129 control arm participants (41.9%) (adjusted odds ratio, 1.02; 95% CI, 0.52-1.99; *P* = .96). In the intervention arm, antidepressant withdrawal symptoms were less severe, and mental well-being was better compared with the control arm; differences were small but significant. There were no significant differences in the other outcomes; 28 of 179 intervention arm participants (15.6%) and 22 of 151 control arm participants (14.6%) experienced adverse events.

**Conclusions and Relevance:**

In this cluster randomized clinical trial of adding internet and telephone support to a practitioner review for possible antidepressant discontinuation, depression was slightly better with support, but the rate of discontinuation of antidepressants did not significantly increase. Improvements in antidepressant withdrawal symptoms and mental well-being were also small. There were no significant harms. Family practitioner review for possible discontinuation of antidepressants appeared safe and effective for more than 40% of patients willing and well enough to discontinue.

**Trial Registration:**

ISRCTN registry Identifiers: ISRCTN15036829 (internal pilot trial) and ISRCTN12417565 (main trial)

## Key Points

**Question:**

Is adding internet and telephone support to a family practitioner review to consider discontinuing long-term antidepressants safe and more effective than practitioner review alone?

**Findings:**

In this cluster randomized clinical trial including 330 enrolled adults, scores on the PHQ-9 depression questionnaire were slightly better in the supported arm after antidepressant discontinuation, as were scores for withdrawal symptoms and mental well-being. However, the proportion of patients achieving discontinuation was 45.5% with support vs 41.9% without, a nonsignificant difference.

**Meaning:**

This study found that a family practitioner review for possible antidepressant discontinuation was safe and effective for more than 40% of patients willing and well enough to discontinue and that remote support provided small additional benefits.

## Teaser

This cluster randomized clinical trial evaluates whether adding internet and telephone support to a family practitioner review to consider discontinuing long-term antidepressant treatment is safe and more effective than practitioner review alone.

## Introduction

Antidepressant use is increasing globally as more people are treated for depression.^1^ However, there is concern about the increasing amount of long-term prescribing in primary care in high-income countries, where more than 10% of adults now take antidepressants for depression.^2,3,4,5,6^ The median antidepressant treatment duration for depression is more than 2 years in the UK^5^ and more than 5 years in the US.^6^ Antidepressants have potentially serious adverse effects,^3,4,7^ and maintenance treatment beyond 6 months after remission, for up to 2 years initially, has been recommended only for patients with a high risk of relapse (recurrent depression, residual symptoms, or severe previous episodes),^8,9^ based on evidence mainly from secondary care.^10^

Maintenance treatment reduces relapse risk for recurrent depression in primary care,^11^ but 35% to 60% of primary care patients recover fully after a first depressive episode^12^ and should not be offered maintenance treatment.^8,9^ Surveys of long-term antidepressant users found that 30% to 50% have no evidence-based indication to continue treatment.^13,14^ Primary care antidepressant treatment may continue without review for years.^15^

A recent placebo-controlled trial found that 44% of primary care patients could discontinue long-term antidepressants without relapsing over 12 months, but significant depressive and withdrawal symptoms occurred in the first 12 weeks after discontinuation.^11^ In routine practice, long-term antidepressant users are often uncertain as to whether they can discontinue safely, and if their practitioners do not actively broach discontinuation, they assume treatment should continue indefinitely.^16^ Systematic reviews^17,18^ have identified only 2 trials prompting family practitioners to consider antidepressant discontinuation, which reported that only 6% to 7% of patients discontinued treatment compared with 8% of patients who discontinued receiving usual, unprompted care.^5,19^ Trials of cognitive behavioral therapy or mindfulness-based cognitive therapy reported discontinuation rates greater than 45%,^17,18^ but these therapies are resource intensive and costly to implement at scale.

Our group developed self-management internet and telephone interventions to support a practitioner treatment review of antidepressant discontinuation^20,21^ and tested them in an open trial compared with an unsupported review. The primary outcome was depression, analyzing for noninferiority, to determine whether the intervention was safe, while antidepressant discontinuation and the other secondary outcomes were analyzed for superiority.

## Methods

### Design

This was a 2-arm, 1:1 parallel-group noninferiority clinical trial, with randomization clustered by family practices, to avoid contamination between arms (trial protocol and statistical analysis plan in Supplement 1).^22^ Between December 1, 2018, and March 31, 2022, 131 UK practices were randomized: 66 intervention practices and 65 control practices, including 238 practitioners. Ethics approval was granted by an independent National Health Service (NHS) research ethics committee (North of Scotland). All participants provided written informed consent. This study was conducted in compliance with the ethical principles of the Declaration of Helsinki^23^ and all International Council for Harmonisation Good Clinical Practice guidelines. This report followed the 2010 Consolidated Standards of Reporting Trials (CONSORT) reporting guideline for cluster randomized trials.

### Randomization

Remote randomization by an independent statistician used computerized sequence generation and online allocation. Minimization balanced social deprivation (dichotomized around the median Index of Multiple Deprivation [IMD] score), practice size (large vs small, dichotomized around 8000 patients), and location (urban vs rural local authority).

### Inclusion Criteria

We included all adult patients taking long-term antidepressants for depression who did not have indications according to the 2009 National Institute for Health and Care Excellence depression guideline^9^ (for >1 year for a first episode of depression or >2 years for recurrent depression), who did not currently have depression or were judged to not be at significant risk of relapse by their general practitioner (GP), and who wished to discontinue treatment.

### Exclusion Criteria

Patients were excluded for risk factors for relapse, including depression (9-item Patient Health Questionnaire [PHQ-9]^24^ score of ≥12 at screening [range, 0-27, where higher scores indicate greater depression]); anxiety (7-item Generalized Anxiety Disorder Questionnaire [GAD-7]^25^ score of ≥10 [range, 0-21, where higher scores indicate greater anxiety]); and suicidal ideas (positive score on the PHQ-9 self-harm question or suicidal thoughts expressed at screening or baseline). Additional exclusions were psychiatric treatment, bipolar disorder, psychosis, substance use, dementia, inadequate English language, no internet access, or another indication for antidepressants besides depression.

### Recruitment, Screening, and Consent

Practices willing to participate identified patients through searching their computerized records and mailed them a participant information sheet with a prepaid reply slip to contact the study team only if they wished to participate or actively decline. Patients responding positively were screened by telephone before baseline assessment, which was initially face to face but was conducted via telephone or online from June 2020 due to COVID-19 restrictions. At baseline, the researcher went over the participant information sheet and sought written consent. All patients were told that we were recruiting people taking long-term antidepressants who were well enough to consider tapering their medication, if appropriate, through review by their GP or nurse practitioner (NP). The participant information sheet outlined the 2 arms, but patients did not know at that point which arm their practice was in to avoid differential recruitment to arms based on their expectations.

A feasibility trial (Work Stream 4 [WS4] of the REDUCE [Reviewing Long-Term Antidepressant Use by Careful Monitoring in Everyday Practice] program) was conducted initially over 12 months, and the definitive trial (WS5) was conducted over 3 years after WS4. In WS4, consent for follow-up was originally for only 6 months. Ethics committee, steering committee, and funder approvals were then given to include WS4 as an internal pilot, as the protocol was unchanged, and it satisfied ACCEPT (Acceptance Checklist for Clinical Effectiveness Pilot Trials) criteria.^26^ Participants in WS4 were then asked to consent to 9- and 12-month follow-up, retrospectively.

### Interventions

In both arms, all patients were able to consult their GP or NP to review their antidepressants, with help from the researcher if necessary in getting an appointment immediately after baseline assessment, which may take some weeks for a nonurgent problem in UK general practice. The control condition therefore represented current best practice, rather than usual care, which may be no active review.^15^ In the intervention arm, in addition to active review, GPs or NPs and patients were given access to the internet support, and patients were offered 3 telephone calls with an NHS Talking Therapies Psychological Well-being Practitioner (PWP). Patients in both arms remained under the care of their GPs or NPs. The number and timing of further consultations for discontinuation were agreed between GPs or NPs and patients.

The patient intervention (a digital intervention prototype called *ADvisor*) was developed using theory, evidence, and qualitative interviews with patients.^20^ It aimed to increase patients’ self-efficacy for discontinuing antidepressants safely, following their preferred ways. It included 8 modules (eAppendix 1 in Supplement 2). Access was recorded to estimate compliance and its association with outcomes.

The practitioner intervention (called *ADvisor for Health Professionals*) was similarly developed using mainly focus groups of practitioners.^21^ It included 7 modules (eAppendix 2 in Supplement 2) and gave examples of hyperbolic tapering schedules (slower, proportional tapering rather than linear) for people with particular difficulty discontinuing,^27^ devised by our team pharmacist (C.F.J). eAppendix 2 in Supplement 2 gives example linear and hyperbolic schedules for sertraline.

Support from PWPs in the intervention arm (eAppendix 3 in Supplement 2) was provided at 3 time points after GP or NP reviews. Call 1 (within 2 weeks; duration, 30 minutes) checked patients’ understanding of the intervention and encouraged confidence in antidepressant tapering. Call 2 (timing agreed with patient; duration, 15 minutes) checked on tapering progress and, when necessary, advised patients to recontact their GP or NP. Call 3 (timing agreed with patient; duration, 15 minutes) explored residual withdrawal symptoms and rehearsed techniques for relapse prevention.

A sample of support calls were audio-recorded, transcribed, and analyzed for fidelity against the guidance provided: 2 sets per PWP randomly selected from the first 3 months and 1 set during the last 3 months to check for drift (eAppendix 3 in Supplement 2).

### Blinding

Blinding of participants was impossible given the open cluster design. Self-completed outcome measures were used to prevent researcher rating bias. Analysis was blind to allocation.

### Outcomes

Outcome measures were collected at baseline and 3-, 6-, 9-, and 12-month follow-up. The primary outcome was the PHQ-9 score at 6 months. We also analyzed PHQ-9 scores over 12 months with repeated-measures generalized linear mixed modeling, allowing for clustering within participants over time and within practices.

The PHQ-9 is familiar in UK primary care^28^ and NHS Talking Therapies.^29^ It measures the symptoms of major depressive disorder from the *Diagnostic and Statistical Manual of Mental Disorders*,^30^ was validated against diagnostic interviews,^24^ has high sensitivity and specificity in UK primary care,^31^ and has sensitivity to change.^32^ The minimal clinically important difference, compared with patients’ ratings of improvement, is 1.7 points.^32^

Secondary outcomes were (1) self-reported antidepressant discontinuation (for ≥2 months) at 6 and 12 months; (2) antidepressant withdrawal symptoms at 3 and 6 months, measured using the Discontinuation Emergent Signs and Symptoms Scale (DESS)^33^; (3) anxiety over 12 months, measured using the the GAD-7^25^; (4) quality of life over 6 and 12 months, measured using the EuroQoL EQ-5D-5L (EuroQoL 5-dimension 5-level questionnaire)^34^ and the Short Form SF-12 (Medical Outcomes Study Short Form 12-item version)^35^; (5) scores on the Warwick-Edinburgh Mental Well-being Scale (WEMWBS)^36^; (6) scores on the Howie Patient Enablement Instrument^37^; (7) scores on the Medical Informant Satisfaction Scale^38^; (8) scores on the Antidepressant Side-Effects Checklist^39^; (9) scores on the Changes in Sexual Function Questionnaires^40^; (10) use of health services to calculate costs, determined with a study-specific questionnaire; and (11) adverse events.

We also administered study-specific questionnaires on sociodemographic characteristics and past history of depression. Race and ethnicity were assessed to determine the representativeness of the patients recruited. Patients were asked to self-report their ethnicity at baseline assessment from a choice of UK census descriptions in the sociodemographic characteristic questionnaire (Bangladeshi, Black Caribbean, Black African, Black other, Chinese, Indian, other Asian Group [“please specify”], Pakistani, White, or other [“please specify”]).

The original sample size was calculated for 90% power, with a 1-sided α of 2.5%, to establish noninferiority within 2 points on the PHQ-9, assuming an SD of 5.4, requiring 155 patients to be followed up in each arm. Assuming an estimated mean cluster size of 3 patients per practice (range, 1-7) and an intracluster correlation coefficient of 0.012 gave a 1.033 cluster design effect.^22^ Anticipating 80% follow-up, we aimed to randomize (155 × 2 × 1.033)/0.8 = 402 patients (201 per arm), from 134 practices (67 per arm).

With approval from our study steering and data monitoring committees, the target sample size was reduced in May 2021 to allow for a correlation observed between baseline and 6-month follow-up scores on the PHQ-9 of *r* = 0.47 (95% CI, 0.26-0.63). (This reduction was done without analyzing changes in the actual values on the PHQ-9 and was blind to allocation to intervention or control arm). The approved revised target was 360 patients, maintaining 90% power.

### Statistical Analysis

A detailed statistical analysis plan was developed prior to the final analysis (Supplement 1). Prespecified complete-case analyses at the patient level were performed using mixed logistic and linear regression models, controlling for baseline values, baseline depression and anxiety scores, previous depressive episodes, declared gender identity, age, employment, housing, educational level, marital status, number of dependents, and practice, which was included as a random effect to allow for clustering. Multiple imputation intention-to-treat (ITT) analyses were performed to explore the effects of missing data. No a priori subgroup analyses were planned, and post hoc analyses were exploratory.

In a noninferiority trial, differences between arms can appear reduced in ITT analysis if compliance is less than 100%. Per-protocol analyses (PPA) were therefore carried out, as well as complier-average causal effect (CACE) analyses to address noncompliance. Compliance in the intervention arm for both was defined as completing the first module of the *ADvisor* intervention within 6 months, as that gave the rationale for attempting discontinuation, and could benefit patients even if they did not log on again. Compliance in the control arm was defined as having an active GP or NP treatment review within 6 months. Calculations for the primary outcome was on the basis of a 1-sided *P* < .025, and calculations for the secondary outcomes were on the basis of a 2-sided *P* < .05.

## Results

A total of 330 participants were recruited (325 eligible for inclusion; 178 in intervention practices and 147 in control practices; mean [SD] age at baseline, 54.0 [14.9] years; 223 [68.6%] women) (Table 1). Of the patients eligible for inclusion and for whom race and ethnicity data were available, 318 of 324 (98.1%) were White, and 6 (1.9%) were of other ethnicities (the self-declared ethnicities within this category are not specified as some were very uncommon and could potentially identify participants); 178 were in intervention practices, and 147 were were in control practices (median, 3 per practice [range 1-14]).

**Table 1.** Baseline Characteristics of Intervention and Control Arms

Characteristic	No. (%)		
	Intervention arm	Control arm	Total
**Practice characteristics**			
No.	66	65	131
IMD score			
1-5 (more deprived)	24 (36.4)	22 (33.8)	46 (35.1)
6-10 (less deprived)	42 (63.6)	43 (66.2)	85 (64.9)
Patient list size^a^			
Small	19 (28.8)	22 (33.8)	41 (31.3)
Large	47 (71.2)	43 (66.2)	90 (68.7)
Location^b^			
Urban	48 (72.7)	48 (73.8)	96 (73.3)
Rural	18 (27.3)	17 (26.2)	35 (26.7)
**Patient characteristics**			
No.^c^	178	147	325
Declared gender identity			
Woman	126 (70.8)	97 (66.0)	223 (68.6)
Man	52 (29.2)	50 (34.0)	102 (31.4)
Other or prefer not to say	0	0	0
Age at baseline, mean (SD), y	54.4 (15.0)	53.5 (14.7)	54.0 (14.9)
Race and ethnicity			
White	176 (98.9)	142 (97.3)	318 (98.1)
Other ethnic group^d^	2 (1.1)	4 (2.7)	6 (1.8)
Missing	0	1	1
Previous depression episodes			
None	69 (38.8)	66 (44.9)	135 (41.5)
1	42 (23.6)	23 (15.6)	65 (20.0)
≥2	67 (37.6)	58 (39.5)	125 (38.5)
Marital status			
Married	101 (56.7)	97 (66.0)	198 (60.9)
Cohabiting	19 (10.7)	15 (10.2)	34 (10.5)
Widowed	12 (6.7)	7 (4.8)	19 (5.8)
Separated	5 (2.8)	3 (2.0)	8 (2.5)
Divorced	15 (8.4)	8 (5.4)	23 (7.1)
Single	26 (14.6)	15 (10.3)	41 (12.6)
Missing	0	2	2
No. of dependents in household			
None	143 (80.3)	123 (83.7)	266 (81.8)
1	7 (3.9)	2 (1.4)	9 (2.8)
2	10 (5.6)	7 (4.8)	17 (5.2)
3	3 (1.7)	1 (0.7)	4 (1.2)
4	9 (5.1)	11 (7.5)	20 (6.2)
5	2 (1.1)	0	2 (0.6)
6	4 (2.2)	3 (2.0)	7 (2.2)
Highest educational qualification^e^			
None	8 (4.5)	4 (2.7)	12 (3.7)
CSE or NVQ level 1	8 (4.5)	12 (8.2)	20 (6.2)
GCSE or O level	32 (18.0)	27 (18.4)	59 (18.2)
A level or BTEC	16 (9.0)	19 (12.9)	35 (10.8)
HNC, HND, or City and Guilds	22 (12.4)	15 (10.2)	37 (11.4)
University degree or higher	67 (37.6)	54 (36.7)	121 (37.2)
Vocational qualification	14 (7.9)	6 (4.1)	20 (6.2)
Other	9 (5.1)	7 (4.8)	16 (4.9)
Missing	2	3	5
Employment			
Full-time work	72 (40.5)	55 (37.4)	127 (39.1)
Part-time work	27 (15.2)	39 (26.5)	66 (20.3)
Permanently sick or disabled	4 (2.3)	1 (0.7)	5 (1.5)
Unemployed	3 (1.7)	1 (0.7)	4 (1.2)
Retired	59 (33.2)	42 (28.8)	101 (31.1)
Student	1 (0.6)	2 (1.4)	3 (0.9)
Homemaker	4 (2.3)	2 (1.4)	6 (1.8)
Voluntary work	2 (1.1)	1 (0.7)	3 (0.9)
Other	6 (3.4)	3 (2.0)	9 (2.8)
Missing	0	1	1
Accommodation			
Owner occupied	141 (79.2)	115 (78.2)	256 (78.8)
Council or housing association	9 (5.0)	9 (6.1)	18 (5.5)
Private rental	19 (10.7)	15 (10.2)	34 (10.5)
Job related	1 (0.6)	0	1 (0.3)
Lives with parents	4 (2.2)	5 (3.4)	9 (2.8)
Other	3 (1.7)	2 (1.4)	5 (1.5)
Missing	1	1	2

Abbreviation: IMD, Index of Multiple Deprivation.

^a^ Practice size was dichotomized around the median list size of 8000 patients.

^b^ Urban or rural location was determined according to the Local Authority Districts 2011 Rural Urban Classification.

^c^ One intervention patient and 4 control patients who consented at screening were subsequently excluded as they no longer met inclusion or exclusion criteria by baseline assessment.

^d^ The self-declared ethnicities within the “Other” category are not specified as some were very uncommon and could potentially identify participants.

^e^ CSE is the Certificate of Secondary Education, a qualification in a specific subject formerly taken by school students aged 14 to 16 years, at a level below O (Ordinary) level. Both the CSE and O level were replaced in 1988 by the GCSE, or General Certificate of Secondary Education. NVQ level 1 is the first level of National Vocational Qualification, a work-based job-specific qualification. A level is the advanced secondary education qualification in a specific subject taken by school students aged 17 to 19 years. BTEC is the Business and Technology Education Council certificate work-based vocational qualification taken after secondary school at older than 16 years. HNC (Higher National Certificate), HND (Higher National Diploma), and City and Guilds are more advanced vocational qualifications.

The CONSORT diagram (Figure) shows that, of 6725 patients invited to participate, 1505 responded (22.4%), 550 (8.2%) positively. Of those invited to participate, 330 (4.9%) were eligible on screening, consented, and were enrolled. After screening but before baseline assessment, circumstances changed for 5 patients so that they no longer qualified at the baseline assessment. The first 52 were WS4 patients (recruited from January to April 2019). The remaining 278 were WS5 patients (recruited from June 2020 to March 2022).

**Figure.**
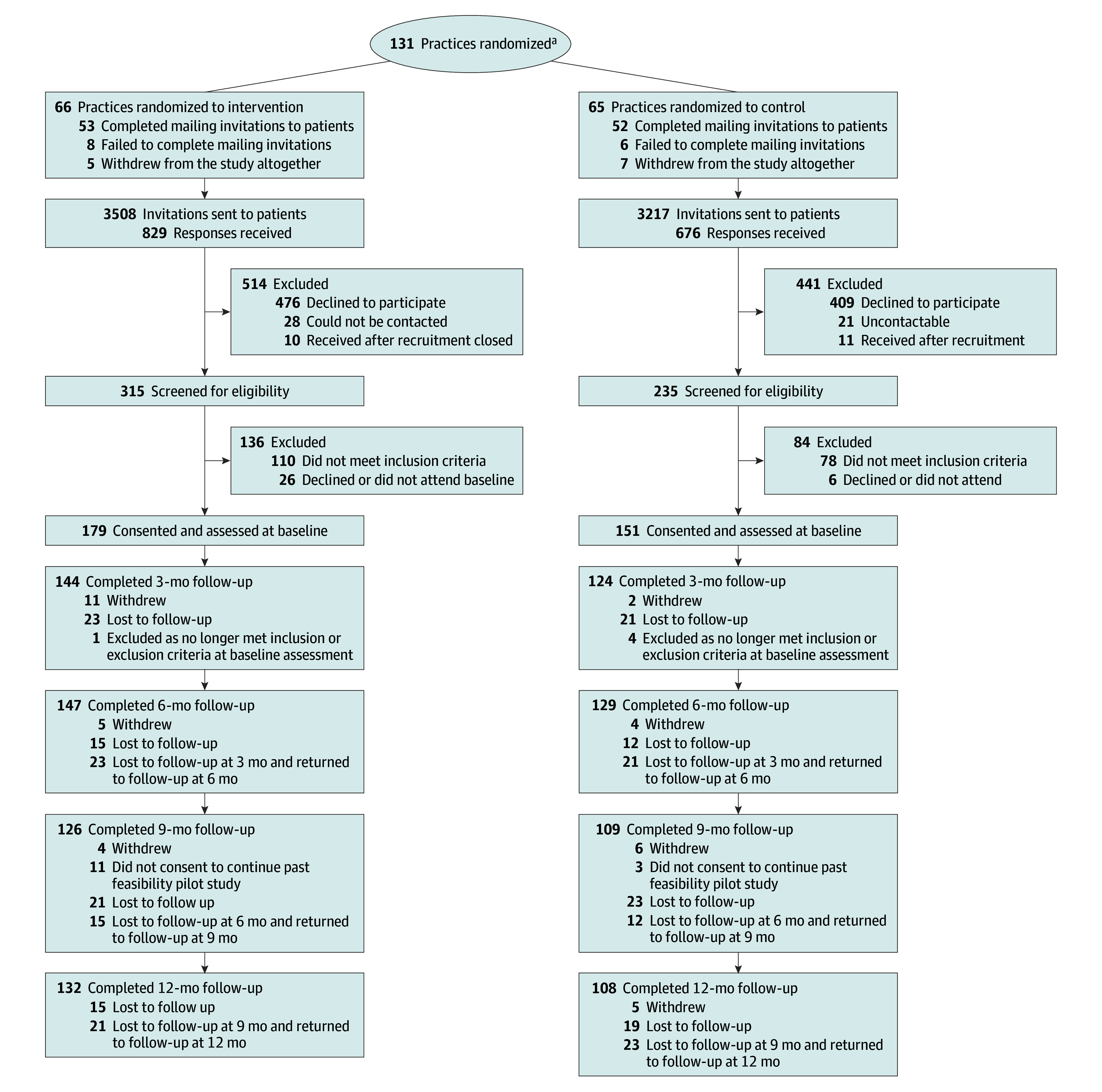
CONSORT Diagram of Practice and Participant Flow Through the Trial ^a^Cluster randomization by practice and minimized by large or small, urban or rural, and deprivation.

### Follow-Up

At 6 months, 276 of the 330 enrolled patients (83.6%) were followed up for the primary outcome: 147 of 179 in the intervention arm (82.1%) and 129 of 151 in the control arm (85.4%) (Figure). At 12 months, 240 of the 330 enrolled patients (72.7%) were followed up: 132 of 179 in the intervention group (73.7%) and 108 of 151 in the control arm (71.5%). This fulfilled our sample size assumption of more than 80% follow-up for the primary outcome at 6 months.

### Baseline Characteristics

Practices were well balanced between arms at baseline (Table 1). Key patient characteristics were also relatively well balanced, with slightly more participants with no previous depression in the control arm, slightly more employed participants and fewer retired people in the control arm, and slightly more married people and fewer single or divorced people in the control arm.

### Compliance

In both arms, 100% of participants were able to undergo a practitioner review. In the intervention arm, 118 of 179 enrolled patients (65.9%) accessed the *ADvisor* intervention, and 144 of 179 (80.4%) received PWP calls.

### Primary Outcome

Table 2 shows changes in scores on the PHQ-9. Mean (SD) scores in the 2 arms were similar at baseline (control, 4.3 [3.2]; intervention, 4.2 [3.6]). The intracluster correlation coefficient was zero. There was a small deterioration in the control arm by 6 months, indicated by an increased mean (SD) PHQ-9 score (intervention, 4.0 [4.3]; control, 5.0 [4.7]; adjusted mean difference, −1.1 [95% CI, −2.1 to −0.1]; *P* = .03). The intervention arm proved noninferior to the control arm in the prespecified complete-case analysis, as the 95% CI for the adjusted mean difference was entirely below the inferiority margin of 2 points. At 6 months, the intervention was seen (post hoc) to be slightly superior, as the 95% CI also was entirely below zero. However, the mean difference of 1 point on the PHQ-9 was smaller than the prespecified threshold for a meaningful difference. In the ITT multiple imputation sensitivity analysis, this difference was attenuated, and while the noninferiority conclusion remained, the intervention no longer appeared superior (adjusted mean difference, −0.9 [95% CI, −1.9 to 0.1; *P* = .08]). The PPA and CACE analyses gave the same inferences as the complete-case analysis.

**Table 2.** Primary Outcome: Depression Measured by the PHQ-9 Scale

Outcome	Intervention (n = 145)^a^	Control (n = 129)	Mean adjusted difference (95% CI)^b^								AOR (95% CI)	*P* value
			Complete-case analysis	*P* value	PPA analysis	*P* value	CACE analysis	*P* value	ITT analysis with 100 imputations^c^	*P* value		
PHQ-9 score at baseline, mean (SD)	4.2 (3.6)	4.3 (3.2)	NA	NA	NA	NA	NA	NA	NA	NA	NA	NA
PHQ-9 score at 6 mo, mean (SD)	4.0 (4.3)	5.0 (4.7)	−1.1 (−2.1 to −0.1)	.03	−1.3 (−2.3 to −0.2)	.02	−1.4 (−2.6 to −0.1)	.04	−0.9 (−1.9 to 0.1)	.08	NA	NA
PHQ-9 score ≥10 at 6 mo (post hoc analysis), No./total No. (%)	16/145 (11.0)	22/129 (17.1)	NA	NA	NA	NA	NA	NA	NA	NA	0.58 (0.24 to 1.41)	.23
PHQ-9 score ≥12 at 6 mo (post hoc analysis), No./total No. (%)	10/145 (6.9)	13/129 (10.1)	NA	NA	NA	NA	NA	NA	NA	NA	0.62 (0.22 to 1.72)	.36

Abbreviations: AOR, adjusted odds ratio; CACE, complier-average causal effect; ITT, intention-to-treat; NA, not applicable; PHQ-9, 9-item Patient Health Questionnaire; PPA, per-protocol analysis.

^a^ Two of the 147 intervention arm patients followed up at 6 months did not complete all the PHQ-9 questions and could not be assigned a total score.

^b^ Models controlled for baseline PHQ-9 score, baseline 7-item Generalized Anxiety Disorder questionnaire anxiety score, declared gender identity, age, employment status, housing type, educational level, marital status, number of dependents, and previous history of depression and included a random effect for practice to allow for the clustered nature of the design.

^c^ An unstructured covariance matrix was used and the imputations were combined using the Rubin rules.

Exploratory post hoc analyses determined the proportions of patients in each arm whose PHQ-9 scores at 6 months were 10 or more or 12 or more, 2 thresholds for possible major depressive disorder.^28^ The proportions were lower in the intervention group for both thresholds, but not significantly (Table 2).

### Secondary Outcomes

Table 3 shows that, at 6 months, more participants in the intervention arm than in the control arm had discontinued antidepressants (66 of 145 [45.5%] vs 54 of 129 [41.9%]), but this difference was not statistically significant (adjusted odds ratio, 1.02 [95% CI, 0.52-1.99]; *P* = .96) (only 145 of the 147 intervention patients provided discontinuation data). This finding was the same in PPA, CACE, and multiple imputation analyses. We explored (post hoc) whether participants were able to reduce their antidepressant dose or completely discontinue it by 6 months. Again, the difference was nonsignificant.

**Table 3.** Main Secondary Outcome: Self-Reported Antidepressant Discontinuation at 6 and 12 Months

Outcome	No./total No. (%) of patients with data at each time point		AOR (95% CI)^a^	*P* value	AOR based on 100 imputations (95% CI)^b^	*P* value
	Intervention arm	Control arm				
Discontinued antidepressants (for >2 mo) at 6 mo	66/145 (45.5)^c^	54/129 (41.9)	1.02 (0.52-1.99)	.96	1.03 (0.55-1.90)	.93
Discontinued (for >2 mo) at 12 mo	46/105 (43.8)	30/79 (38.0)	1.24 (0.62-2.47)	.54	1.45 (0.76-2.74)	.26
Patients at 12 mo who had restarted antidepressants after 6 mo	10/42 (23.8)	11/33 (33.3)	NA	NA	NA	NA
Discontinued antidepressants between 6 and 12 mo	13/58 (22.4)	6/43 (14.0)	NA	NA	NA	NA
Discontinued or reduced the dose of antidepressant by 6 mo (post hoc analysis)	108/145 (74.5)^c^	87/129 (67.4)	0.97 (0.39-2.39)	.95	NA	NA

Abbreviations: AOR, adjusted odds ratio; NA, not applicable.

^a^ Models controlled for baseline 9-item Patient Health Questionnaire score, baseline 7-item Generalized Anxiety Disorder questionnaire anxiety score, declared gender identity, age, employment status, housing type, educational level, marital status, number of dependents, and previous history of depression and included a random effect for practice to allow for clustering.

^b^ An unstructured covariance matrix was used and the imputations were combined using the Rubin rules.

^c^ Two of the 147 intervention arm patients followed up at 6 months did not answer the question on discontinuation of antidepressants.

Table 4 shows the remaining outcomes analyzed using repeated measures over 6 or 12 months. There was no evidence of a treatment by time interaction for depression over 12 months. Over 6 months, DESS antidepressant withdrawal symptom scores were significantly lower in the intervention arm. The difference at 3 months was due to a reduction from baseline in the intervention arm. Over 12 months, WEMWBS mental well-being scores were also significantly better in the intervention arm. There were no significant differences in anxiety, quality of life, enablement, satisfaction, antidepressant adverse effects, service use, or costs.

**Table 4.** Secondary Outcomes in Intervention and Control Arms

Outcome	Mean (SD) value										Adjusted mean difference over 6 mo (95% CI)^c^	*P* value	Adjusted mean difference over 12 mo (95% CI)^c^	*P* value
	Intervention arm					Control arm								
	Baseline (n = 178)^a^	3 mo (n = 144)	6 mo (n = 147)	9 mo (n = 126)	12 mo (n = 132)^b^	Baseline (n = 147)^a^	3 mo (n = 124)	6 mo (n = 129)	9 mo (n = 109)	12 mo (n = 108)^b^				
PHQ-9 depression symptom score	4.2 (3.6)	4.3 (4.2)	4.0 (4.3)	4.7 (4.8)	4.2 (4.2)	4.3 (3.2)	5.0 (4.1)	5.0 (4.7)	5.7 (4.5)	4.8 (4.7)	NA	NA	−0.7 (−1.4 to 0.01)	.05
GAD-7 anxiety symptom score	3.2 (2.8)	3.7 (4.0)	3.2 (3.8)	3.7 (4.4)	3.4 (3.9)	3.4 (3.4)	3.8 (3.3)	3.8 (3.9)	4.1 (4.1)	3.9 (3.8)	NA	NA	−0.2 (−0.8 to 0.4)	.53
DESS withdrawal symptoms scale	12.6 (7.3)	10.8 (7.3)	11.8 (8.5)	NA	NA	12.6 (7.8)	12.6 (7.6)	12.8 (8.6)	NA	NA	−1.6 (−2.9 to −0.3)	.02	NA	NA
WEMWBS mental well-being score	50.3 (9.4)	NA	49.8 (11.2)	NA	48.6 (12.4)	51.0 (9.3)	NA	48.3 (10.3)	NA	47.3 (13.4)	NA	NA	2.2 (0.2 to 4.1)	.03
EuroQoL EQ-5D-5L quality of life score	0.74 (0.11)	NA	0.75 (0.13)	NA	0.74 (0.13)	0.74 (0.11)	NA	0.72 (0.12)	NA	0.71 (0.13)	0.049 (−0.002 to 0.099)	.06	0.022 (−0.030 to 0.075)	.36
SF-12 score	0.84 (0.14)	NA	0.83 (0.16)	NA	0.83 (0.15)	0.81 (0.16)	NA	0.78 (0.17)	NA	0.80 (0.16)	0.041 (−0.012 to 0.093)	.12	0.010 (−0.060 to 0.080)	.74
Patient Enablement Instrument score	NA	NA	1.1 (1.0)	NA	1.3 (1.0)	NA	NA	1.3 (1.0)	NA	1.5 (1.1)	NA	NA	−0.2 (−0.4 to 0.01)	.06
MISS distress relief score	NA	NA	42.5 (14.1)	NA	40.4 (12.8)	NA	NA	41.3 (13.3)	NA	38.4 (13.0)	NA	NA	−0.02 (−3.0 to 3.0)	.99
MISS communication comfort score	NA	NA	17.0 (5.0)	NA	16.2 (4.8)	NA	NA	17.3 (4.8)	NA	15.5 (4.6)	NA	NA	−0.1 (−0.9 to 1.2)	.81
MISS rapport score	NA	NA	46.8 (13.3)	NA	44.6 (13.2)	NA	NA	46.8 (12.4)	NA	41.1 (14.7)	NA	NA	−0.3 (−2.7 to 3.2)	.87
MISS compliance intent score	NA	NA	17.1 (4.7)	NA	16.8 (4.6)	NA	NA	17.1 (4.5)	NA	15.3 (4.5)	NA	NA	0.4 (−0.6 to 1.4)	.48
ASEC total score	7.5 (5.0)	NA	6.2 (6.2)	NA	5.9 (5.5)	7.5 (5.3)	NA	7.1 (5.5)	NA	6.1 (5.1)	−0.6 (−1.5 to 0.2)	.14	−0.9 (−2.0 to 0.3)	.12
CSFQ total score	33.6 (11.2)	NA	33.9 (12.2)	NA	31.6 (14.7)	35.2 (11.4)	NA	33.8 (12.9)	NA	31.7 (14.4)	NA	NA	0.6 (−2.2 to 3.3)	.69
Contacts with primary care health services	NA	NA	NA	NA	6.1 (5.1)	NA	NA	NA	NA	6.7 (5.1)	NA	NA	−0.6 (−1.8 to 0.5)	.80
Contacts with secondary care health services	NA	NA	NA	NA	1.7 (2.6)	NA	NA	NA	NA	1.9 (2.6)	NA	NA	−0.2 (−0.8 to 0.4)	.87
Total costs of health service contacts	NA	NA	NA	NA	£596 (£1663); $757 ($2112)	NA	NA	NA	NA	£669 (£922); $850 ($117)	NA	NA	–£69 (−£77 to £207); –$88 (−$98 to $263)	.82

Abbreviations: ASEC, Antidepressant Side-Effect Checklist; CSFQ, Changes in Sexual Functioning Questionnaires; DESS, Discontinuation Emergent Symptoms and Signs; EuroQoL EQ-5D-5L, EuroQol 5-dimension 5-level questionnaire; GAD-7, 7-item Generalized Anxiety Disorder questionnaire; MISS, Medical Informant Satisfaction Scale; NA, not applicable (not all outcomes were analyzed at each time point, as specified in the protocol); PHQ-9, 9-item Patient Health Questionnaire; SF-12, Medical Outcomes Study Short Form 12-item version; WEMWBS, Warwick-Edinburgh Mental Well-being Scale.

^a^ One intervention participant and 4 control participants were excluded immediately after randomization because they either met the exclusion criteria or no longer met the inclusion criteria at the baseline assessment; thus, baseline measures were collected for 325 instead of 330 participants.

^b^ The difference in costs between the arms was estimated using bootstrapping, with 2000 resamples with replacement (Bank of England exchange rate £1 = $1.27 on January 23, 2024).

^c^ Models controlled for baseline score of the variable being analyzed, employment status, housing type, educational level, marital status, dependents, declared gender identity, age, past history of depression, baseline PHQ-9 depression score, and baseline GAD-7 anxiety score and included a random effect for practice to allow for clustering.

### Adverse Events

Altogether 69 adverse events were reported, by 28 of 178 intervention arm participants (15.7%) and 22 of 147 control arm participants (15.0%) (*P* = .86). Eleven serious adverse events were recorded, for 2 intervention arm and 5 control arm patients (4 experienced 2 events each; *P* = .15). Nine serious events were hospital admissions unrelated to the trial, and 2 serious events were serious adverse reactions. One intervention arm patient was admitted to a psychiatric unit for relapse of an anxiety disorder and withdrew from the study. One control arm patient was referred urgently to psychiatric outpatients due to expressed suicidal ideas but was not admitted and remained in the study.

### Outcomes Associated With Use of the Online Intervention

eAppendix 1 in Supplement 2 shows analyses of outcomes associated with patients’ recorded use of *ADvisor*. The PHQ-9 scores were slightly higher, but not significantly, among those who consulted the module on dealing with withdrawal symptoms compared with those who did not. Antidepressant discontinuation was higher among those who completed modules on keeping well, values and goals, and moving forward and lower among those completing modules on thinking about antidepressants, dealing with withdrawal, and worrying about stopping, but the difference was significant only for worrying about stopping (odds ratio, 0.13 [95% CI, 0.04-0.41]).

### Fidelity of Psychological Well-Being Practitioner Support Calls

In total, 35 calls were audio-recorded and transcribed for analysis to check fidelity against guidance provided (eAppendix 3 in Supplement 2). In 100% of calls, the PWPs asked how tapering was going, administered the PHQ-9, and asked about use of the intervention, the 3 essential elements.

## Discussion

### Main Findings

Provision of internet and telephone support proved noninferior to an unsupported practitioner review in terms of the primary outcome, depressive symptoms. However, there was no significant difference in the rate of discontinuation of antidepressants between arms. We found, post hoc, a statistically significant benefit in terms of depressive symptoms in the prespecified complete-case analysis, but the difference was small, within the prespecified threshold for a meaningful difference, and not significant in an ITT multiple imputation sensitivity analysis.

The support also reduced withdrawal symptoms and conserved mental well-being, but, again, the differences were small and of uncertain clinical benefit. Reduced withdrawal symptoms may have been due to increasing patient awareness of their characteristics through the online education, so fewer symptoms were considered to be due to withdrawal. However, these benefits, although small at the individual level, tended to reduce calls on health services and therefore may be cost-effective at a population level.

Only approximately 15% of patients in each arm reported adverse events, and there was only 1 serious adverse reaction in each arm, so discontinuation is safe to attempt in primary care, as long as patients are monitored for relapse and treatment can be quickly restarted.

### Limitations

This study has some limitations. We recruited 330 patients, slightly short of the (revised) target sample of 360, but we had sufficient power to address the primary outcome with precision. However, only 72.7% of patients were followed up at 12 months, which reduced the power to exclude longer-term differences in depression and antidepressant discontinuation. Only 8.2% of patients approached wanted to take part, and only 4.9% were enrolled. However, a very low response rate to cold-calling invitations is common in trials, as patients understandably have concerns about untested interventions. A similarly low rate of willingness to attempt antidepressant discontinuation was found in a questionnaire survey of patients’ intentions in routine care.^41^ Participants could not be blinded to receiving support, and outcomes were self-reported, so some of the differences might be due to response bias.

Two-thirds of participating practices were in areas with above-average IMD scores, and our findings may not be generalizable to more deprived areas. Only 1.9% of participants were from racial and ethnic minority groups compared with 19% of people in the 2021 UK census,^42^ and our intervention needs wider testing (we have developed an Urdu version).

Antidepressant discontinuation in both arms greatly exceeded the 6% to 7% rate found in previous trials of simply prompting GP treatment reviews.^5,19^ This finding is probably because patients in our study had to consent to attempt discontinuation, whereas in the previous studies, all patients receiving long-term treatment were eligible, including many who were unwilling to discontinue treatment.^5,19^ General practitioner treatment reviews in the UK may also have improved over time as more guidance on antidepressant discontinuation has been published.^43,44^

## Conclusions

This cluster randomized clinical trial found that internet and telephone support did not significantly increase antidepressant discontinuation but provided small improvements in depression, antidepressant withdrawal symptoms, and mental well-being. An active family practitioner review for possible discontinuation of inappropriate long-term antidepressant treatment proved to be safe and effective in both arms for more than 40% of patients willing to discontinue. To our knowledge, this is the first study to demonstrate that facilitating discontinuation is possible at scale without providing resource-intensive psychological therapy.^17,18^ The proportion of patients who achieved success was similar to the proportion who did not relapse in the placebo arm of the previous placebo-controlled trial of withdrawing maintenance treatment in primary care.^11^

The findings of this study suggest that an active family practitioner review for possible antidepressant discontinuation should be promoted, and more research should be carried out on motivating both patients and practitioners to attempt discontinuation when appropriate, providing information on the possible benefits as well as the relapse risk. Replication of our research is needed, taking steps to improve intervention effectiveness as well as engage people from deprived areas and racial and ethnic minority groups to widen the interventions’ generalizability. In addition to analyzing the interventions’ cost-effectiveness, we are conducting a process evaluation to identify the more promising elements of the interventions to provide possible improvements.

## Notes

Supplement 1.
Trial Protocol and Statistical Analysis Plan

Supplement 2. **eAppendix 1.** The Patient Online Intervention “*ADvisor*”
eReferences 1.
**eAppendix 2.** The Practitioner Online Intervention “*AdvisorHP”*
eReferences 2.
**eAppendix 3.** PWP Telephone Support

Supplement 3.
Data Sharing Statement
