# Supplementary material for: Internet and Telephone Support for Discontinuing Long-Term Antidepressants: The REDUCE Cluster Randomized Trial
Source: JAMA Netw Open. 2024 Jun 24;7(6):e2418383. doi: 10.1001/jamanetworkopen.2024.18383 (PMC11197448; doi:10.1001/jamanetworkopen.2024.18383)
Supplement: Supplement 1. — Trial Protocol and Statistical Analysis Plan [file jamanetwopen-e2418383-s001.pdf]

Medicine

UNIVERSITY OF Southampton

REDUCE (REVIEWING LONG TERM ANTIDEPRESSANT USE BY CAREFUL MONITORING IN EVERYDAY PRACTICE)

PROTOCOL for WORK STREAM 5: REDUCE RANDOMISED CONTROLLED TRIAL

Chief Investigator: Prof Tony Kendrick MD FRCGP FRCPsych (Hon)
Professor of Primary Care
Primary Care & Population Sciences
University of Southampton
Aldermoor Health Centre
Southampton SO16 5ST

Sponsor: **University of Southampton**

ERGO Ref: **49190**

IRAS Ref: **266517**

Funder: **NIHR Programme Grant for Applied Research (PGfAR)**
Ref: **RP-PG-1214-20004**

ISRCTN no: **12417565**

Dates: **01/11/2019 – 31/10/2022**

**Protocol Version**	**Protocol date**	**Signature of CI**
1.0	06.06.2019	
1.1	26.06.2019	
1.2	12.07.2019	
**1.3**	**04.09.2019**	
**1.4**	**28.11.2019**	
**1.5**	**01.05.2020**	
**1.6**	**10.06.2020**	
**1.7**	**20.7.2020**	
**1.8**	**19.08.2020**	
**1.9**	**06.05.2021**	
**1.10**	**22.06.2021**	

SERIOUS ADVERSE EVENTS

All serious adverse events (SAEs) will be reported to the Programme Manager at REDUCE@soton.ac.uk (Tel: 02380 591754) and Sponsor - University of Southampton within 24 hours of the local site becoming aware of the event. We will use the Southampton CTU's SAE Non-CTIMP Form, which asks for the nature of the event, date of onset, severity, corrective therapies given, outcome, causality (i.e. unrelated, unlikely, possible, probably, definitely) and expectedness.

The Chief Investigator will assign the causality and expectedness of the event and the term should be in accordance with the latest version of MedDRA and grades given in accordance with the NCI CTCAE v4.03. Additional information will be provided as soon as possible if the event has not resolved at the time of reporting.

Definitions

Adverse Event (AE): any untoward medical occurrence in a participant or clinical study participant which does not necessarily have a causal relationship with study treatment or participation.
An AE can therefore be any unfavourable and unintended sign (including an abnormal laboratory finding), symptom, or disease temporally associated with the study treatment or participation (regardless of causality assessments).

Serious Adverse Event (SAE) is any untoward medical occurrence or effect that:

- **Results in death**
- **Is life-threatening** – *refers to an event in which the participant was at risk of death at the time of the event; it does not refer to an event which hypothetically might have caused death if it were more severe*
- **Requires hospitalisation, or prolongation of existing hospitalisation**
- **Results in persistent or significant disability or incapacity**
- Other important medical events***.

*'life-threatening' in the definition of 'serious' refers to an event in which the patient was at risk of death at the time of the event; it does not refer to an event which hypothetically might have caused death if it were more severe.

**Hospitalisation is defined as an inpatient admission, regardless of length of stay, even if the hospitalisation is a precautionary measure for continued observation. Hospitalisations for a pre-existing condition, including elective procedures that have not worsened, do not constitute an SAE.
***Other important medical events May also be considered serious if they jeopardise the participant or require an intervention to prevent one of the above consequences.

Note: It is the responsibility of the PI or delegate to grade an event as 'not serious' (AE) or 'serious' (SAE).

Causality

A complete assessment of the causality must always be assessed by a medically qualified doctor who is registered on the delegation of responsibility log; this will usually be the Principal Investigator at each University (below).

Relationship	Description	Event Status
**Unrelated**	There is no evidence of any causal relationship	Not related to treatment
**Unlikely**	There is little evidence to suggest there is a causal relationship (e.g. the event did not occur within a reasonable time after administration of the study treatment). There is another reasonable explanation for the event (e.g. the participant's clinical condition, other concomitant treatment).	Not related to treatment
**Possibly**	There is some evidence to suggest a causal relationship (e.g. because the event occurs within a reasonable time after administration of the study treatment). However, the influence of other factors may have contributed to the event (e.g. the participant's clinical condition, other concomitant treatments).	Related and expected SAE/ Related and unexpected SAE
**Probably**	There is evidence to suggest a causal relationship and the influence of other factors is unlikely.	Related and expected SAE/ Related and unexpected SAE
**Definitely**	There is clear evidence to suggest a causal relationship and other possible contributing factors can be ruled out.	Related and expected SAE/ Related and unexpected SAE

In terms of event status; **Not related to treatment** would highlight that the SAE is not related to the trial treatment. **Related and expected** SAE would signify that the SAE is related to the trial treatment and is expected (according to the list of expected events listed in the protocol). **Related and unexpected SAE** would be classified as an SAE which is related to the trial treatment and is unexpected in terms of the events listed in the protocol.

In the case of discrepant views on causality between the Investigator and others, the SCTU will classify the event as per the worst case classification I and where applicable the Ethics Committee will be informed of both opinions within the required timelines.

Expectedness

Expectedness assessments are made against the list of expected events below:

6.4.1 Expected Adverse Events:

Mild antidepressant withdrawal symptoms	Likely to occur in around half of patients reducing and stopping their antidepressants. If mild enough to be tolerated by patient, and short-lived (up to two weeks), these do not need reporting as AEs.
Moderate to severe withdrawal symptoms	If severe or prolonged withdrawal symptoms occur, requiring restarting of antidepressant, please report as related and expected AE.
Relapse of depression or anxiety disorder	May occur in 10-20% of patients over the one year follow-up, requiring restarting of antidepressant. Please report as related and expected AE.

The nature or severity of should be considered when making the assessment of expectedness. If these factors are not consistent with the current information available then the AE should be recorded as 'unexpected'.

Contents

SERIOUS ADVERSE EVENTS 2

Definitions 2

Causality 3

Expectedness 3

MAIN STUDY CONTACTS 8

SPONSOR 8

CO-APPLICANTS, COLLABORATORS, AND RESEARCH TEAM 8

Research team, University of Southampton 9

Research team, University of Liverpool 9

Research team, University of Hull 9

FUNDER 10

PROTOCOL INFORMATION 10

LAY SUMMARY 10

BACKGROUND INCLUDING OVERVIEW OF THE REDUCE PROGRAMME 11

REDUCE PROGRAMME AIM 11

REDUCE PROGRAMME OBJECTIVES 11

REDUCE WORK STREAMS 12

WS1. Systematic review of interventions facilitating antidepressant cessation (October 2016 to September 2017). 12

WS2. Qualitative interviews with patients and practitioners (October 2016 to September 2017). 12

WS3. Co-production of internet-supported practitioner and patient interventions (October 2017 to September 2018). 12

WS4. Feasibility RCT, to assess acceptability, recruitment, and outcome measures (October 2018 to October 2019). 13

REDUCE programme benefits to patients and the NHS 13

PROTOCOL FOR WORK STREAM 5: REDUCE randomised controlled trial 13

Aim 13

Design 13

Randomisation 13

Inclusion criteria 14

Exclusion criteria 14

Patient recruitment 14

Consent procedure 15

Intervention arm 15

Control arm 16
Numbers of eligible patients expected per practice 16
Sample size calculation 17
Baseline telephone screening 17
Outcome measures 18
Data collection 18
Blinding 18
Telephone follow-up where necessary will be carried out by a research assistant in a different University to the recruiting University, blind to practice allocation, who will advise the patients on first contact not to reveal which arm of the trial they are in. Any inadvertent unblinding will be recorded and reported. The trial RAs will also obtain information from medical records, but at the end of the study, in order not to unblind them to practice allocation during patient follow-up. 18
Discontinuation of antidepressants 18
Quality of life 19
Mental wellbeing 19
Antidepressant withdrawal symptoms 19
Antidepressant side effects 19
Patient satisfaction 20
Beliefs about antidepressants 20
Collective efficacy 20
Enablement 20
Costs 20
Consent and data collection summary 20
STATISTICAL ANALYSIS 21
HEALTH ECONOMIC EVALUATION 22
PROCESS EVALUATION 22
Qualitative 23
Quantitative 23
Psychological process measures 23
ETHICAL CONSIDERATIONS 24
PROGRAMME STEERING COMMITTEE 24
INDEPENDENT DATA MONITORING COMMITTEE 24
SAFETY OF PARTICIPANTS IN THE TRIAL 24
WITHDRAWAL FROM THE STUDY 25
CONFIDENTIALITY 25

INDEMNITY 25

DATA HANDLING 25

MONITORING 25

RECORD RETENTION AND ARCHIVING 26

PUBLICATION AND DATA SHARING POLICY 26

REFERENCES 26

MAIN STUDY CONTACTS

Chief Investigator: Professor Tony Kendrick
Primary Care and Population Sciences, Faculty of Medicine, University of Southampton
Tel: 02380 501790
Email: ark1@soton.ac.uk

Programme manager: Wendy O'Brien
Primary Care and Population Sciences, Faculty of Medicine, University of Southampton
Tel: 02380 591754
Email: W.Obrien@soton.ac.uk

Study Coordination:
For general study and clinical queries e.g. participant queries, study supplies, data collection etc., please contact by email in the first instance: REDUCE@soton.ac.uk

SPONSOR

The University of Southampton is the research sponsor for this study. For further information regarding sponsorship conditions, please contact the Head of Research Governance at: Research Governance Office, University of Southampton, Room 4079, Building 37, Highfield Campus, Southampton SO17 1BJ
Tel: 023 8059 5058
Fax: 023 8059 5781
Email: rgoinfo@soton.ac.uk

CO-APPLICANTS, COLLABORATORS, AND RESEARCH TEAM

Prof Michael Moore Principal Investigator, University of Southampton

Prof Paul Little, University of Southampton

Prof Geraldine Leydon, University of Southampton

Professor Guiqing Lily Yao, University of Leicester

Dr Beth Stuart, University of Southampton

Dr Adam Geraghty, University of Southampton

Professor Gareth Griffiths, Southampton Clinical Trials Unit

Prof Carl May, London School of Hygiene and Tropical Medicine

Prof Mark Gabbay Principal Investigator, University of Liverpool

Dr Joanna Moncrieff, University College London

Prof Glyn Lewis, University College London

Prof Simon Gilbody, University of York

Prof Una Macleod (Principal Investigator, University of Hull)

Dr Chris Johnson, NHS Greater Glasgow and Clyde

Dr Susan Collinson, PPI Representative, London

Margaret Bell, PPI representative, Portsmouth

Bryan Palmer, PPI representative, Southampton

Research team, University of Southampton

Dr Hannah Bowers, Research Fellow

Ms Riya Tiwari Senior Research Assistant

Ms Amy Din Senior Research Assistant
--
Dr Shihua Zhu, Health Economist

Sonja Poore, Administrative Assistant

Research team, University of Liverpool

Tasneem Patel, Researcher

Dr Helen Page, Researcher

Daniel Lawrence, Administrative assistant

Research team, University of Hull

Researcher: Dr Mahboobeh Haji Sadeghi, Research Fellow

Amy Porter, Administrative assistant

FUNDER

This study is funded by an NIHR Programme Grant for Applied Research (PGfAR), ref: RP-PG-1214-20004.

PROTOCOL INFORMATION

This protocol describes the REDUCE work stream 5 and provides information about procedures for entering participants. The protocol should not be used as a guide for the treatment of other non-study participants; every care was taken in its drafting, but corrections or amendments may be necessary. These will be circulated to investigators in the study, but sites entering participants for the first time are advised to contact REDUCE@soton.ac.uk at the University of Southampton to confirm they have the most recent version.

LAY SUMMARY

There is considerable concern about increasing antidepressant use in England. GPs are writing more than 60 million prescriptions a year, to around 1-in-10 adults. Some people need long-term antidepressants to stop them getting depressed, but a third-to-a-half could possibly stop them without relapsing. However, stopping is not always easy. Some patients may experience withdrawal symptoms, while others may be fearful that they will experience them. Withdrawal symptoms can include anxiety and depression, which are usually temporary, but can feel similar to the reasons why patients first started antidepressants. So understandably, some people restart their antidepressant quickly. Others are reluctant to try stopping because they feel well on medication, they are afraid their symptoms may come back, and they feel that there is too much at stake to take the risk of stopping.

Taking antidepressants long-term exposes patients to the risks of side-effects. Common side effects of antidepressants include changes in weight, changes in sleep, and changes in libido or sexual functions. Less commonly, some patients develop bleeding from the stomach or intestine, and the use of antidepressants in people over 65 is associated with an increase in the risks of falls, seizures, and strokes. Some patients also complain that they feel emotionally numb on antidepressants. These are all good reasons for proposing that the drugs should not be continued long-term unless there is a good reason for taking them.

People taking antidepressants have told us their GPs rarely review them and just give repeat prescriptions. Studies show that when GPs do review patients on long-term antidepressants and recommend that they could begin to stop taking them, only 1-in-14 is able to stop. Patients can be fearful and cessation can be tricky, so GPs and Nurse Practitioners (NPs) who prescribe antidepressants need to develop individual cessation strategies for each patient, and be able to offer them sustained support, especially in the first few weeks or months.

The NHS funded six-year **REDUCE** (**RE**viewing long term anti**D**epressant **U**se by **C**areful monitoring in **E**veryday practice) research programme aims to identify feasible, safe, effective, and cost-effective ways of helping patients taking long-term antidepressants taper off and stop treatment, when appropriate. This Work Stream 5 (WS5) of the REDUCE programme aims to determine the effectiveness of online (Internet) interventions which support practitioners and guide patients on coming off antidepressants, together with psychological practitioner telephone calls to support the patients. This work stream draws on a previous systematic review and qualitative synthesis of the existing literature on antidepressant cessation carried out in work stream 1; analyses of qualitative interviews and focus groups carried out with patients and practitioners respectively on the difficulties of antidepressant cessation in work stream 2; the development of the intervention using ‘think-aloud’ interviews with patients and practitioners in work stream 3; a feasibility randomised trial in work stream 4; the lived experience of our patient and public involvement (PPI) advisors; and our team's research and development expertise.

WS5 is a fully powered randomised controlled trial (RCT), which aims to assess the effectiveness of the Internet and telephone interventions. It will take 36 months to complete, starting in October 2019. We will recruit 402 patients (201 randomly allocated to the intervention arm and 201 controls), from 134 general practices over 15-18 months,

and follow them up for 12 months. We will assess the effectiveness of the interventions in terms of reductions in antidepressant use in the absence of worsening of depression, and assess patients' and practitioners' use of the interventions (automatically recorded by the Southampton 'LifeGuide' software used for the Internet guidance).

A qualitative process evaluation will be conducted through open-ended interviews with 15-20 patients and 15-20 practitioners in each arm. We will explore why the interventions were effective or not, depending on the results. We will also look at the use of the telephone support provided by psychological practitioners (PWPs), and any technical support needed for patients to be able to use the internet intervention. The interviews will be recorded and typed out (transcribed) for 'thematic analysis', which is line by line examination of the interview transcripts to identify themes among the reactions of the patients and practitioner involved in the trial.

BACKGROUND INCLUDING OVERVIEW OF THE REDUCE PROGRAMME

Antidepressant prescriptions have risen steadily year on year since 1990, because GPs have been prescribing longer and longer courses (Moore et al, 2009; Kendrick et al, 2015a), and the average length of treatment is now more than two years. Some people need long-term antidepressants to prevent relapse, but surveys suggest 30-50% have no evidence-based indication for long-term use (Cruikshank 2008). However stopping is not easy, due to withdrawal symptoms including anxiety and mood changes which feel similar to the reason why treatment was started in the first place (Fava, 2015). Patients on long-term treatment are often given repeat prescriptions and are reviewed only infrequently (Middleton, 2011; Sinclair, 2014).

Taking antidepressants long-term exposes patients to the risks of side-effects. Common side effects of antidepressants include changes in weight, changes in sleep, and changes in libido or sexual functions (Ferguson, 2001). Less commonly, some patients develop bleeding from the stomach or intestine, and the use of antidepressants in people over 65 is associated with an increase in the risks of falls, seizures, strokes (Coupland, 2011). Around 1 in 2 patients on antidepressants feel emotional blunting or numbness (Goodwin 2017). So the drugs should not be continued long-term unless there is a good reason for taking them.

Antidepressants constitute a substantial proportion of the NHS drug budget: 2.5% in 2010 (Ilyas & Moncrieff, 2012) and the costs of unnecessary treatment include appointments for medical or nursing reviews. The cost of GP consultations for depression exceeded £30m in 2008 (Independent Research Service of the House of Commons Library), in addition to the cost of the 53 million antidepressant prescriptions per year, of around £300m, so substantial savings could be made if significant numbers of long-term users were to discontinue.

Many patients are dissatisfied with this situation, and would like help to stop long-term treatment. However, GPs often lack experience in reducing antidepressant medication flexibly, and GP advice to taper and stop treatment is not often successful. Prompting GPs to review patients eligible for stopping treatment was tested in a trial in the Netherlands and found to be ineffective, with only 6% of patients discontinuing in the intervention group, and 8% in the control group (Eveleigh 2014; Eveleigh 2015). Similarly, an uncontrolled trial of pharmacist-prompted GP review of long-term users in Scotland resulted in only 7% stopping (Johnston 2012). Therefore it appears that without a specific intervention addressing issues with patient and practitioner behaviours, many patients will continue antidepressants unnecessarily. Practitioners need guidance to provide support for tapering and stopping treatment, and patients need 24 hour support.

REDUCE PROGRAMME AIM

To identify feasible, safe, effective, and cost-effective ways of helping patients taking long-term antidepressants taper and stop treatment when appropriate.

REDUCE PROGRAMME OBJECTIVES

1. Conduct a systematic review of interventions used to help patients successfully stop antidepressant treatment.
2. Identify factors promoting or inhibiting treatment cessation, through interviews with patients, and focus groups with practitioners.

3. Develop an internet-supported CBT-based intervention to support treatment tapering and cessation, through co-design and co-production with practitioners and patients.
4. Determine the effectiveness of the intervention through a randomised controlled trial, and estimate cost-effectiveness from a health/social service perspective.
5. Build a translational framework addressing how the intervention should be delivered, including overcoming practitioner and patient related barriers, to facilitate implementation.

REDUCE WORK STREAMS

WS1. Systematic review of interventions facilitating antidepressant cessation (October 2016 to September 2017).

The existing literature was searched to inform the planning of our intervention. Papers were identified through comprehensive searches (including Medline/Embase/WHO clinical trials registry/ CINAHL/ PsycINFO/OpenGrey). A thematic synthesis of previous qualitative research was performed, and a narrative review of quantitative studies plus meta-analysis of two pairs of studies: a pair on using cognitive behaviour therapy (CBT) to help with stopping antidepressants, and a pair on using mindfulness based cognitive therapy (MBCT).

Qualitative synthesis of barriers and facilitators (from 21 studies) identified nine important factors: psychological/physical capabilities; perceptions of antidepressants/depression; fear factor; motivators/goals; the doctor as a navigator to discontinuation; information to support decision-making; significant others; support from other professionals. A systematic review of managing discontinuation (15 studies, 12 with analysable results, with meta-analysis possible for 2 pairs of RCTs) showed no difference in cessation rates between CBT + taper (95%) and clinical management + taper (91%), but a lower risk of relapse with CBT. No difference was found in relapse rates between MBCT + taper (achieving cessation in >70%), and maintenance antidepressants.

WS2. Qualitative interviews with patients and practitioners (October 2016 to September 2017).

Planning the intervention was also informed by 19 individual qualitative patient interviews, and focus groups with 24 GPs, 7 practice nurses, and 6 psychological practitioners (PPs), in the Wessex region. Thematic analysis was used, checking agreement between analysts, and discussing emerging themes with the whole team.

The following themes emerged: patient themes: beliefs about medication, fear of relapse and withdrawal symptoms, patient-practitioner interactions (uncertainty about whether to ask to come off), influence of family and friends; practitioner themes: supporting patients rather than making decisions for them, assessing risk and managing expectations, organisational factors, beliefs, need for psychological tools in the Internet intervention.

WS3. Co-production of internet-supported practitioner and patient interventions (October 2017 to September 2018).

The interventions were developed based on the literature reviews in WS1 and qualitative interviews with patients and practitioners in WS2, plus theories of behaviour change (Michie) and normalisation process theory (May). Prototype web-based interventions for patients and practitioners were created in LifeGuide, and we used qualitative 'think-aloud' interviews with 15 patient and 19 practitioner participants, to explore whether they were acceptable, interesting, persuasive, and feasible. Thematic analysis of the think aloud interviews was used to identify improvements in the interventions, iteratively, between rounds of interviews. The feedback from users provided information about the positive aspects of the interventions and highlighted areas where improvements could be made. Further iterations of the interventions were then made at stages during WS3 and recruitment for interviews ceased when the feedback was no longer resulting in the need for further changes to the interventions.

The patient intervention includes on-line advice delivered using University of Southampton LifeGuide software on: why try to reduce treatment, dealing with withdrawal symptoms, when to seek help, alternatives to drug treatment, and guided self-help tools based on CBT, MBCT, and acceptance and commitment therapy (ACT).

A separate web-based LifeGuide intervention for the prescribing GPs and nurses includes information and advice on which patients can safely try tapering off treatment, broaching the subject, tapering regimes (including individual antidepressant drug tapering schedules), and schedules for follow-up patient reviews. Telephone support is also provided by a psychological practitioner (PP) for an average of one hour per patient over three calls.

As a result of the think-aloud interviews, two interventions were developed that were informed by the specific needs and preferences of their intended users. Following the completion of interviews, the interventions went through a rigorous testing phase to ensure that all the digital elements worked as intended. The intervention was then ready for use in the feasibility study.

WS4. Feasibility RCT, to assess acceptability, recruitment, and outcome measures (October 2018 to October 2019).

WS4 is a pilot feasibility randomised controlled trial (RCT), to assess the acceptability of the Internet and telephone interventions, the recruitment of practitioners and patients, and the acceptability of the planned outcome measures. It will take 13 months to complete, by the end of October 2019, having started in October 2018.

We recruited 52 patients (27 patients randomly allocated to the intervention arm and 25 controls), from 14 general practices over 6 months, and are currently following them up for 6 months. We are assessing ease of practice and patient recruitment; loss of patients to follow-up; the ease of use and acceptability of our outcome questionnaires; and participants' use of the interventions (recorded by ‘LifeGuide’ software automatically).

REDUCE programme benefits to patients and the NHS

If successful, REDUCE will lead to reductions in inappropriate treatment of patients, including the costs of drugs, and the cost of monitoring patients, in the absence of any worsening of patients’ depression (primary outcome).

PROTOCOL FOR WORK STREAM 5: REDUCE randomised controlled trial

Aim

The aim of the REDUCE randomised controlled trial (RCT), which is work stream 5, to start in October 2019, is to evaluate the online and telephone interventions to support patients and practitioners in the reduction of antidepressant medication for depression where appropriate.

Design

The trial will be cluster randomised by participating general practices. Whole practices will be randomised to the intervention or control arms, rather than randomising individual patients, in order to avoid contamination between arms (the inadvertent application of the intervention to control patients).

Randomisation

Randomisation will be computerised and carried out independently by the Southampton Clinical Trials Unit (SCTU). We will use the statistical technique of ‘minimisation’ to balance practice size (large/small), location (urban/rural), and social deprivation (dichotomised around the median Index of Multiple Deprivation (IMD) score). There is a random element to the minimisation algorithm, and so we might not expect perfect balance to the randomisation.

Inclusion criteria

Our aim is to include patients who are taking long-term antidepressant treatment, which is not indicated according to the NICE depression guideline (NICE, 2009). We will therefore include all consenting:

- patients on treatment for more than 1 year for a first episode, and
- patients treated for more than 2 years for a recurrent episode, who are:
- no longer depressed or judged to be at significant risk of relapse

Exclusion criteria

The significant risk factors for relapse are:

- current significant depressive symptoms on the PHQ-9 (see below) despite antidepressant treatment
- current significant anxiety symptoms on the GAD-7 (see below)
- current suicidal ideas (see below)
- current psychiatric outpatient or inpatient treatment for depression

In addition to the above criteria increasing the risk of relapse, the following will also be exclusion criteria:

- bipolar disorder, comorbid psychosis, substance use, or dementia as a primary diagnosis
- spoken or written English language inadequate to take part in interviews or complete questionnaires
- another indication for taking antidepressants, e.g. neuropathic pain

Patient recruitment

Potential patient participants will be approached in two ways:

(i) through practice records database searches, and
(ii) opportunistically in general practitioner (GP) or nurse practitioner (NP) consultations

All eligible patients identified by searches will be actively approached in both intervention and control arms, to avoid the risk of selection bias inherent in relying only on opportunistic recruitment by practitioners.

Practice computerised medical records databases will be searched using 'Read' diagnostic and symptom codes for depressive diagnoses and symptoms, together with British National Formulary chapter codes for antidepressants taken over the previous two years. Standardised searches were developed during the WS4 feasibility RCT, for the two main practice computer systems SystmOne and EMIS, which can be given to participating practices. Participating GPs will check the lists of potential participants against the inclusion and exclusion criteria, to ensure all are suitable to be invited to take part.

Mail-out packs will be sent by post, emailed or sent by SMS to patients. The pack will include an invitation letter from the GP, the Participant Information Leaflet (PIL), and a reply slip for the patients to complete indicating whether they are interested in taking part. We will use the Docmail digital mail-out facility where possible. Interested patients will be asked to contact the REDUCE team directly using the reply slips (in Freepost envelopes) or by email. If patients do not contact the research team then the team will have no knowledge of their names or addresses, thus maintaining patient confidentiality.

Eligible patients may also be invited to consider taking part within a GP or NP consultation for depression. Patients will be given a pack by the practitioner in person, emailed or sent via an SMS which includes the GP invitation Letter, PIL and reply slip. This should be returned directly to the research team using a Freepost envelope or by email if the patient is interested in discussing possible involvement in the trial. Again, there will be no contact between the research team and potential patients unless the patients initiate it.

Those patients who return reply slips to the research team indicating a willingness to discuss possible participation will be contacted by a member of the research team by telephone and screened for the exclusion criteria. This will involve asking a standard set of yes/no questions and administration of the PHQ-9 for depressive symptoms and the GAD-7

for anxiety symptoms (see below). Patients will be reminded of the information provided to them in the PIL sent with the GP invitation letter, and if they have no exclusion criteria they will be asked for a convenient time to meet with a member of the research team either in person or remotely, using Skype or MS TEAMS, telephone, or another communication platform.

We will ask the participating general practice if they would like to send a reminder invitation to participate letter, to participants who do not respond to the first invitation to participate. We think that some participants may not be ready to taper their anti-depressants when they receive the first invitation, for reasons that may be transient, for example too close to Christmas. As people's situations and personal circumstances fluctuate, a follow up letter may be received at a time that the participant considers more appropriate for tapering.

Consent procedure

The researcher will arrange to meet the patient, either remotely, or at their GP practice, or at their home if they prefer, to go over the consent procedure. If the patient gives verbal and then subsequent written consent to take part, the researcher will conduct a baseline assessment. Information given at the point of consent will be the same in both arms. All patients will be told we are recruiting people who have been taking antidepressants for more than a year for a first episode, or more than two years for a recurrent episode, with a view to working out how to help them reduce their medication if appropriate, with the advice of their practice GP or nurse (see patient information leaflet and consent form).The information leaflet will outline the two different approaches but not in detail and potential participants will not know to which arm their general practice has been randomised. This is to avoid differential rates of consent to the two arms based on patients' opinions of the intervention or procedures involved in each.

Patients who do not wish to consent to take part at that point for reasons which may be temporary (i.e. they do wish to try to reduce and stop taking their antidepressant, but not at that time due to current life stresses, recent life events, or the timing of upcoming events etc.), will have the option of consenting to be re-contacted after three months, to be asked again if they wish to participate at that later point.

Having once consented to take part in writing on this initial basis, and having undergone baseline assessment, patients will then be given further information about the details of the procedures in the arm to which their practice has been randomised. In the intervention arm, patients will be given advice and support to log on and engage with their web-based support (see below). After looking at the on-line ADvisor for patients, they will be asked to arrange to consult with their GP/NP either remotely or in person to discuss coming off antidepressants, including agreeing a time to start tapering the dose, and a first follow-up appointment for review.

The researcher will book the patient in for their first Psychological Practitioner (PP) appointment, timed within the first two weeks of their GP appointment (see below). In the control arm, patients will be asked to arrange to consult with their GP/NP either remotely or in person to discuss whether or not they should try coming off their antidepressants. Whether or not they start to taper will be a matter for them to agree with their GP or prescribing nurse.

Intervention arm

The practitioner intervention (called 'ADvisor for Health Professionals' as it gives advice about <u>A</u>nti<u>D</u>epressants) includes Internet modules on: Why reduce; Broaching the subject; When to start tapering; Reduction schedules for individual antidepressants; Dealing with withdrawal symptoms; Dealing with relapse; ADvisor for patients (a summary); and printable pages on antidepressant reduction regimes and sections of ADvisor for patients to recommend patients consult.

The patient intervention (called 'ADvisor for Patients') includes Internet modules on: Reducing and stopping (introduction to website); How to reduce antidepressants; Thinking about antidepressants (their effects and why lifelong treatment may not be necessary); Dealing with withdrawal symptoms; I'm worried about stopping; Keeping well; Thinking about what you value in life; and Moving forward.

In the intervention arm practices, the GPs/NPs will be given access to the on-line ADvisor for practitioners, and induction to the study, which will be either practice-based or on-line. They will receive an introduction to ADvisor, and education on best practice in the supervision of antidepressant tapering and cessation, focussing on the differences between withdrawal symptoms and relapse, and the management of withdrawal symptoms.

The number and timing of subsequent GP/NP consultations during tapering and following drug cessation will be left to the participating GPs/NPs to agree with the patients on an individual basis.

In addition to the ADvisor Internet modules and GP/NP consultations, the following telephone support will be provided to patients in the intervention arm by a trained psychological practitioner (PP):

- Call 1 (0-2 weeks), for 30 minutes: to check the patient's understanding of the ADvisor intervention and encourage confidence in going through the tapering and cessation process
- Call 2 (4-6 weeks), for 15 minutes: to ask the patient how tapering is going and whether they are following the schedule, and where necessary, to advise the patient to discuss any issues with tapering with their GP
- Call 3 (timing agreed with patient), for 15 minutes: to ask the patient about any residual withdrawal symptoms and go over techniques to help with relapse prevention.

A sample of 10-20 telephone calls per practitioner will be audio-recorded in the first three months, and again halfway through the trial, to check for fidelity against the telephone support guise.

Control arm

In the control arm, participating practices will be informed that the recruited patients are potentially eligible for tapering off antidepressants. Their electronic medical records will be flagged and patients will be asked to make an appointment either remotely or in person as part of usual care to see their GPs/NPs for a review, but they will not be trained in best practice in tapering, unlike practitioners in the intervention arm.

Alerting the control arm practices to the potential eligibility of patients for tapering off antidepressants, will result in some patients tapering and ceasing treatment. Some patients would have discontinued treatment anyway, as part of usual care. This will be permitted within the trial. Our power calculation for the secondary outcome of antidepressant discontinuation in the main trial assumes a 7% discontinuation rate in the control practices, which is the rate found in previous studies of simply prompting GPs to review patients potentially eligible for discontinuation (Johnson et al, 2012, Eveleigh 2015).

Numbers of eligible patients expected per practice

An analysis of GP recording of depressive symptoms and diagnoses in the Clinical Practice Research Datalink (CPRD) between 2003 and 2013 (Kendrick 2015a) indicates an expected annual incidence of new cases of depression of around 1%, or 70 patients in an average 7,000-patient practice. We know from the CPRD data that around 60% of these (40 patients) will be prescribed antidepressants (Kendrick 2015b), of whom we estimate 36% (14 patients) will still be taking antidepressants 12 months after diagnosis (beyond the duration recommended by NICE (2009) of six months after remission – unpublished CPRD data, Kendrick), so we estimate 14 incident cases per practice per year will be eligible to try tapering and ceasing treatment.

In addition to these incident cases, our CPRD data (Kendrick 2015a) indicate an annual rate of prevalent (recurrent) cases of around 4% (or 280 patients per average practice), of whom around 75% (210 patients) will be prescribed antidepressants (Kendrick 2015b). Of these around half (105 patients) will have taken them for longer than two years (Johnson 2012) of whom we estimate (conservatively) that 20% (21 patients) will not be at significant risk of relapse (Cruikshank 2008), and could therefore also be advised to try coming off treatment according to NICE guidelines (2009).

This means at least 35 patients in the average practice will be eligible for possible tapering off treatment, so recruitment of three patients per practice can be achieved even if only 8.5% of eligible patients agree to participate in

the study. Eveleigh (2014) recruited 146 from 45 significantly smaller Dutch practices (3.2 per practice), with a participation rate of 15% of those approached, so we are confident this is feasible.

We were able to recruit 52 patients from 14 practices (a mean of 3.7 patients per practice) in our WS4 feasibility RCT, reinforcing our confidence in being able to recruit at least three patients per practice for WS5.

Sample size calculation

We originally calculated that, to have 90% power, with a one-sided alpha of 2.5%, to establish non-inferiority in terms of depressive symptoms within 2 points (MCID) on the PHQ-9 at 6 months (SD 5.4), we needed 155 patients followed up in each arm. Assuming a variable cluster size of between 1 and 7 per practice (mean 3) and an intra-cluster correlation coefficient (ICC) of 0.012 (from the HTA THREAD trial of treating mild to moderate depression in primary care, Kendrick 2009) gave a 1.033 cluster design effect (based on a coefficient of variation of 1.5/3=0.5, using the formula in Eldridge 2006). Anticipating 20% do not comply with the intervention and/or are lost to follow up, we needed to randomise (155x2x1.036)/0.8=402 patients (201 per arm), from 134 practices (67 per arm).

However we found (in May 2021) a significant correlation between baseline and follow-up values for the primary outcome (the PHQ-9 score at 6 months) of 0.47 (95% confidence interval 0.26 to 0.63). Assuming conservatively that this correlation remains 0.26 or greater until the end of follow-up, the necessary target sample size to give 90% power will be reduced by a deflation factor of $1\text{-}0.26^2$ (Borm, 2007) which means we can achieve 90% power to address the research question with a total sample size of 375 patients recruited, given the other parameters in the original sample size calculation remain unchanged. If the current level of correlation of 0.47 persists until the end of follow-up, the sample size needed for 90% power would be reduced to a total recruited of 313 patients. We assumed that the level of correlation was likely to end up somewhere between 0.26 and 0.47 and, with the agreement of the Funder the NIHR, and the Sponsor, the University of Southampton, the target sample size was therefore reduced to 360 patients (May 2021).

As no significant changes were made to the intervention or trial procedures following the feasibility study, we propose the feasibility WS4 study is accepted as an internal pilot RCT, and that the data collected within can be taken forward for inclusion within the WS5 definitive RCT. The ACCEPT acceptance checklist for clinical effectiveness pilot trials (Charlesworth et al, 2013) will be used to assess whether the feasibility trial data can be included in the main trial. The decision about whether to use the data will be a matter for our Programme Steering Committee to judge, informed by our Independent Data Monitoring Committee (see below).

Baseline telephone screening

Patients will be assessed by telephone and excluded if they have any of the exclusion criteria listed above. Exclusion criteria include significant depressive symptoms despite antidepressant treatment, defined as a score of 12 or more on the Patient Health Questionnaire (PHQ-9) (Kroenke 2001) completed over the telephone. The PHQ-9 is a self-complete questionnaire taking approximately three minutes. It measures nine core symptoms of depression based on Diagnostic and Statistical Manual (DSM-IV) criteria and has high sensitivity and specificity in UK primary care (Gilbody 2007).

Patients will also be excluded if they have significant anxiety at baseline, i.e. a score of 10 or more on the 7-item Generalised Anxiety Disorder scale (GAD-7), also completed over the telephone. The GAD-7 is a brief screening instrument for anxiety also validated in primary care (Spitzer 2006). Though originally developed to detect GAD, it also has good sensitivity and specificity for panic, social anxiety, and post-traumatic stress disorders (Kroenke 2007).

If patients score above 0 (i.e. 1, 2 or 3) on the 9th question of the PHQ-9, about suicide/self-harm, they will be excluded from participating, and this information will be relayed to their GP immediately for them to discuss this, preferably with their permission. The information may be relayed without their permission if necessary, after discussion between the researcher, the principal investigator, and the patient.

If patients are excluded at baseline screening due to either a total PHQ-9 depression score of 12 or more, or a score of 1 or more on the self-harm question 9 of the PHQ-9, or a total GAD-7 anxiety score of 10 or more, and they wish to be reconsidered for inclusion at a later date, they may consent to being recontacted in three months to be re-screened. At re-screening, they may be included in the study if those exclusion criteria no longer apply.

The other exclusion criteria will be addressed through telephone screening of patients against a checklist of: current psychiatric outpatient or inpatient treatment for depression (yes/no); comorbid psychosis, substance use or dementia as a primary diagnosis (yes/no); spoken or written English language inadequate to take part in interviews or complete questionnaires (yes/no); and another indication for taking antidepressants, e.g. neuropathic pain (yes/no). These exclusion criteria are permanent and would not qualify the patient for consideration again after three months.

Baseline measures will also include a bespoke sociodemographics questionnaire, questions on internet experience, work status, comorbidities, a questionnaire on health service resource use (looking back over the preceding 6 months), and quality of life measured using both the EQ-5D-5L and SF-12 (see details below; again over the preceding 6 months).

Outcome measures

The primary outcome for the main trial (WS5) will be the PHQ-9 score at 6 months, and secondary outcomes will include discontinuation of antidepressants; mental wellbeing; antidepressant withdrawal symptoms; antidepressant side effects; patient satisfaction; patient enablement; quality of life; and costs.

Data collection

The outcome measures will be collected at baseline, 3, 6, 9, and 12 months (see table below). Data collection at baseline will be face to face or remotely, but data will be entered on-line using i-survey (University of Southampton secure online survey system) where possible. Data collection at 3 and 9 months will be through either online i-survey, or postal follow-up, with one reminder after two weeks, and subsequent remote, face-to-face or telephone follow-up, to obtain the outcomes for patients who do not complete i-survey or return their questionnaires by post. Postal questionnaires will be accompanied by an explanatory letter requesting return of completed questionnaires within two weeks if possible. Data collection at 6 and 12 months will be remote or face to face. Patient participants will be given a £10 gift voucher for their time at the 6 month assessment.

We will ask participants when we screen them if they are happy to receive texts. If participants are happy to disclose their mobile number we will keep in regular contact with them using the University of Southampton text messaging service. Text messages will be sent to remind participants about appointments and completing online questionnaires. Relevant information on consultations at the practice and use of services outside the practice will also be extracted from patients' medical records by practice staff at the end of the study, assuming they have given consent for this.

Blinding

Telephone follow-up where necessary will be carried out by a research assistant in a different University to the recruiting University, blind to practice allocation, who will advise the patients on first contact not to reveal which arm of the trial they are in. Any inadvertent unblinding will be recorded and reported. The trial RAs will also obtain information from medical records, but at the end of the study, in order not to unblind them to practice allocation during patient follow-up.

Discontinuation of antidepressants

Discontinuation of antidepressants will be deemed to have occurred once the patient has stopped taking them for two months. Determining discontinuation after six months will allow tapering and cessation to take up to four months. In our experience, after two months, withdrawal symptoms will have mostly gone, and mood problems will have re-emerged if they are going to. We think patients who decide to resume treatment are unlikely to wait even one month.

Quality of life

We anticipate that successful tapering and cessation of treatment will improve patients' quality of life (QoL) by reducing the adverse effects associated with taking antidepressants, along with the other burdens of unnecessary treatment including GP appointments and pharmacy visits.

We will measure QoL using the EuroQol-5D (EQ-5D)-5L. The EQ-5D-5L is the measure favoured by NICE in determining cost-effectiveness when developing its clinical guidelines. The EQ-5D-5L includes five dimensions: mobility, self-care, usual activities, pain/discomfort, and anxiety/depression, each scored on five levels (no problems, slight problems, some problems or severe problems and extreme problems). It is an improved version of the original EQ-5D-3L which was developed to reduce ceiling effects experienced with the EQ-5D-3L. We will apply the UK value set and scoring algorithm to translate EQ-5D-5L scores to utility scores (Devlin 2016).

We will also assess quality of life using the Medical Outcomes Study-derived measure of functional health status, the 12-item Short Form (SF-12) (Ware 1996), from which utilities can be derived using the SF-6D (Brazier 2004). One reason for using both measures is that the SF-6D may be more sensitive to changes in quality of life related to mild depression than the EQ-5D-5L (Peasgood 2012). We also do not know which measure will be more sensitive to changes in quality of life resulting from relief from antidepressant side effects on the one hand, or withdrawal symptoms on the other, resulting from antidepressant discontinuation. We therefore propose to use the SF-12 in addition to the EQ-5D-5L in WS5 and explore the implication of quality of life gained or loss in our study population in a sensitivity analysis.

Mental wellbeing

A recent systematic review (Brazier 2014) found that the EQ-5D and SF-6D, while capturing changes in quality of life due to mental and physical illness, were less good at capturing other important aspects, including control, autonomy and choice; self-perception; belonging; activity; and hope. We will therefore use the Warwick-Edinburgh Mental Wellbeing Scale (WEMWBS) (Stewart-Brown 2011) as an additional secondary outcome measure, since this measures both subjective experiences of happiness and life satisfaction (the 'hedonic perspective'); and positive psychological functioning, good relationships with others and self-realisation (the 'eudaimonic perspective'). The latter includes capacity for self-development, positive relations with others, autonomy, self-acceptance and competence.

Coming off long-term antidepressants might improve these aspects of wellbeing, since side effects can include emotional blunting which should be reduced, and a greater sense of autonomy and self-acceptance might result from not having to rely on medication. WEMWBS has been shown to be responsive to change with a range of mental health interventions in specialist and community populations (Maheswaran 2012).

Antidepressant withdrawal symptoms

These will be measured using the Discontinuation Emergent Signs and Symptoms Scale (DESS), a brief self-report measure on which participants can indicate the presence of, and changes in, 43 possible antidepressant withdrawal symptoms (Rosenblaum 1988). Withdrawal symptoms will be measured at 3 and 6 months, asking patients to rate their presence looking back over the period since recruitment.

Antidepressant side effects

These will be measured at baseline, 6, and 12 months (for those patients still taking antidepressants at follow-up), using the Antidepressant Side-Effects Check-list (ASEC) developed by Aitchison, as part of the GENDEP research project (http://gendep.iop.kcl.ac.uk/results.php). It asks participants to rate the presence of 21 possible side effects and also includes open questions for other symptoms not listed, and demonstrates good agreement between self-report and psychiatrists' ratings (Uher 2009).

At baseline, 6 and 12 months, we will also use the Changes in Sexual Functioning Questionnaire (CSFQ-C), a 14-item self-rating instrument including five domains of sexual functioning, which has been shown to be reliable and valid in both clinical and research settings (Clayton 1997; Keller 2006).

Patient satisfaction

We will assess this using the 29-item 'Medical Interview Satisfaction Scale' (MISS-29) which was developed in the USA to assess patient satisfaction with individual doctor-patient consultations and has been shown to be valid and reliable in UK primary care (Meakin 2002). We will adapt it to rate patient satisfaction at the 6-month follow-up, asking patients to look back over their consultations with the GPs/PNs for advising on tapering and cessation of antidepressants.

Beliefs about antidepressants

A bespoke questionnaire asking for patients' beliefs about antidepressants, and their cessation, developed by a Southampton PhD student Rachel Dewar-Haggart, will be administered at the face-to-face assessments at baseline, 6 months and 12 months. This will enable a mediator analysis of possible effects of changes in patients' beliefs on changes in antidepressant use.

Collective efficacy

At the 3-month assessment point, we will administer the collective efficacy questionnaire (Band 2019) which is a measure of the strength of support for discontinuing antidepressants which a participant perceives among their important friends and family. The importance of this support as a possible moderator of the success of the intervention

Enablement

We will also use the Patient Enablement Instrument (PEI) that is designed to capture patients' ability to understand the nature of their problems and cope with their illness (Howie 1998). It was developed in primary care to be completed by the patient after a consultation. We will adapt it to rate patient enablement at the 6-month follow-up, looking back over the whole period of the intervention, as was done successfully in the ATEAM trial (Little 2008), when the adapted measure proved sensitive to change.

Costs

A societal perspective will be taken for the health economics analysis. Bespoke questionnaires, developed in light of the WS1 systematic review and WS2 qualitative interviews, and modified in light of the experience of using them in WS4, will be used to collect data on health and social service resource use; personal out of pocket spending; and time off work.

In addition, a review of patients' computerised GP records will be conducted by practice staff to extract any additional health service usage including medication, primary care consultations, outpatient appointments, A&E attendances, and hospital admissions. All items will be costed using appropriate data (e.g. British National Formulary, Personal Social Service Research Unit (PSSRU) and NHS reference costs), with informal care costed at minimum wage level.

The resource use questionnaire will be administered face to face with patients at baseline, 6 and 12 months, asking them to look back over the previous 6 months at each point. The case note review will be done by general practice staff looking back over the previous six months from the six-month follow-up point, and again from the end of the 12 months follow-up.

Consent and data collection summary

**Measure**	**Reply slip**	**Screening (telephone)**	**Baseline (Remote/face to face)**	**3 months (postal or on-line)**	**6 months (Remote/face to face)**	**9 months (postal**	**12 months (Remote/face to face)**

						**or on-line)**	
Consent to be contacted about the study	√						
Inclusion/exclusion criteria		√					
Consent to participate			√				
Sociodemographics and past history of depression questionnaire			√				
Depression (PHQ-9)		√	√	√	√	√	√
Anxiety (GAD-7)		√	√	√	√	√	√
Suicidal ideas		√	√	√	√	√	√
Discontinuation of antidepressants (for at least 2 months, by 6 months)					√		
Quality of life (EQ-5D, SF-12)			√	√	√	√	√
Wellbeing (WEMWBS)			√		√		√
Withdrawal symptoms (DESS)			√	√	√		
Antidepressant side effects (ASEC, CSFQ-C) (if taken)			√		√		√
Satisfaction (MISS-29)					√		√
Enablement (PEI)					√		√
Questionnaires on use of services, use of antidepressants, sickness absence, and beliefs about antidepressants			√		√		√
Collective efficacy questionnaire				√			

STATISTICAL ANALYSIS

Intention to treat (ITT) analyses at patient level will be performed, using mixed logistic/linear regression models, controlling for baseline values, stratification variables, and potential confounders as appropriate. Practice will be modelled as a random effect to allow for the clustering of patients within practices. Patterns of missing data will be explored, sensitivity analysis used to explore the impact of missingness, and imputation of missing data will be used. In a non-inferiority trial where some patients do not comply with treatment as randomised, the difference between the arms can appear reduced and the groups look more similar, leading to the incorrect conclusion of non-inferiority. A per protocol (PP) analysis would analyse individuals based on their compliance with treatment as randomised, excluding non-compliant participants, giving a more conservative estimate of effect for non-inferiority (Piaggio 2006).

However, the exclusion of some participants after randomisation can potentially lead to bias. Therefore we will present both ITT and PP analyses.

We will also undertake a complier-average causal effect (CACE) analysis, another approach to dealing with noncompliance that compares compliant participants in the intervention group, with those in the control group whose characteristics are similar enough to the intervention group compliers to suggest they too would have complied with the intervention, given the opportunity to do so (Angrist 1996, Dunn 2005). Compliance for these analyses in the intervention arm will be defined as completing the first session of the LifeGuide programme within 6 months of recruitment (anticipating 90% plus). The first session will have information about antidepressant treatment, the rationale for attempting withdrawal, and how withdrawal should be attempted under supervision. We would expect patients to benefit from that session even if they do not log on again. Compliance in the control arm will be defined as having consulted the GP/PN to have their antidepressant treatment reviewed within the 6-month follow-up period. For the primary outcome we will report the analyses based on all three approaches and interpret the findings cautiously in light of any differences between approaches that may emerge. This analysis will form the core of the publication of the trial results. No interim analyses will be performed.

For secondary outcomes the analyses will use a similar modelling approach to that set out for the primary outcome, with mixed logistic/linear regression models as appropriate, with a random effect for Practice, controlling for baseline values, stratification variables, and potential confounders as appropriate. Discontinuation of antidepressants will be evaluated at the 6 month time point. All other secondary measures will be analysed using a repeated measures approach with measures clustered within patients over time in addition to the clustering of patients within practice. The models will control for stratification variables, and potential confounders as appropriate and, where a baseline measure for the outcome is available, will for the baseline value, too.

A detailed statistical plan will be developed and discussed with the Independent Data Monitoring Committee and Programme Steering Committee (see below).

HEALTH ECONOMIC EVALUATION

For the full trial, our proposed economic evaluation will be taken from an NHS and PSS perspective with a sensitivity analysis from a societal perspective. The outcome will be expressed as incremental cost per point improvement in the PHQ-9 clinical outcome, incremental cost per discontinuation of antidepressants, and incremental cost per quality adjusted life year (QALY) gained (cost utility analysis). Case note review at 6 and 12 months will augment the 6-monthly patient reports of health and social service resource use. All items will be costed using appropriate data (e.g. PSSRU, NHS reference costs), with informal care costed at minimum wage level. The primary analysis will be at 12 months. Personal costs will include patient and carer time off work, personal expenses, use of Internet, and travel. Itemised resource usage will be weighted by associated unit costs and aggregated over 12 months. Quality adjusted life years (QALYs) will be estimated by the area under the curve approach.

A generalised linear mix model will be used to estimate the differences in costs and QALYs (using both EQ-5D and SF-6D utilities), adjusting for baseline characteristics including socio-economic deprivation and internet use. Where appropriate we will estimate incremental cost-effectiveness ratios (ICERs). We will estimate mean values and 95% percentiles using non-parametric bootstrapping, and use these to estimate cost-effectiveness acceptability curves (CEACs). Major assumptions in the costing and quality of life analysis will be tested through sensitivity analyses. The decision analytic model developed during WS2 will be updated by the full trial evidence to extrapolate the cost-effectiveness beyond the trial period covering the potential risk of recurrence if the intervention proves to be effective in terms of improvements in QALYs up to 12 months. The health economics analysis will be published.

PROCESS EVALUATION

The objective of the process evaluation in WS5 is to identify, characterise and explain factors likely to inform practitioner and patient behaviour change, to inform a robust implementation plan for the appropriate

discontinuation of antidepressants in clinical practice, through identifying and solving potential problems likely to inhibit the translation of the lessons of the REDUCE Trial into the everyday management of depression in primary care.

Qualitative

The qualitative process study will use the same procedures as in the feasibility trial, interviewing 15-20 purposively sampled patients in each arm (or more if needed for saturation), and 15-20 practitioners in each arm, remotely or face to face, for up to 60 minutes. Interviews will be transcribed and themes identified through inductive analysis using constant comparison. We will interpret these in the light of Normalization Process Theory and use this to construct a taxonomy of factors affecting both the conduct of the trial, and its normalization potential beyond the trial. We anticipate where NPT does not characterise and explain data collected, we will identify constructs within the Consolidated Framework for Implementation Research (Damschroder 2009), and Michie's (2011) Theoretical Domains Framework. We will then be able to show how taxonomy components are explicable by reference to known mechanisms of embedding and integration in practice. The analysis will enable construction of an implementation framework of barriers and facilitators (patient and health system factors) that need to be taken into account in the design and delivery of the normalized intervention in practice, which we will publish.

Interviews will be semi-structured, focused on patient and practitioner experiences, but also informed by the 16 items developed to identify features of a complex intervention likely to be 'intervention killers' in everyday practice and operationalised in toolkit form at www.normalizationprocess.org (Murray 2010).

Individual patient interviews will explore in-depth user experiences of engaging with the intervention for a prolonged period, including:

- What worked well and what could have worked better
- Perspectives on mode of delivery and content, to gauge usability and understanding
- Burden of treatment from the patient's perspective

Individual practitioner interviews will explore their views on:

- Negotiating the decision to taper off treatment with their patients
- Their role as a GP/NP/PWP in terms of supporting/negotiating appropriateness of cessation
- Support needs in practice
- Ways to optimise implementation of cessation in routine practice, focusing on individual issues
- Follow-up monitoring of patients undergoing treatment tapering and cessation

The interviews will be transcribed and the data subject to inductive analysis using constant comparison to identify themes. We will interpret these in the light of Normalization Process Theory and construct a taxonomy of factors likely to affect the uptake and implementation of the intervention, and patient outcomes.

Quantitative

We will examine the effects of baseline patient characteristics (e.g. demographics; prior internet experience, clinical characteristics) on engagement with the intervention and outcomes. Automatic data collection by LifeGuide will assess engagement (usage patterns), choice of self-management activities (e.g. cognitive exercises), progress and outcomes, and we will employ multi-level modelling to investigate how these relate to outcomes in mediation analyses.

Psychological process measures

We will evaluate key psychological processes to find which elements are most important in predicting the success or otherwise of the intervention. These will include measures of patients' confidence, expectations and self-efficacy related to being able to reduce and stop their antidepressants. The questions will be taken from validated scales included in a psychometrically tested questionnaire developed by Rachel Ryves, a PhD student in our department at Southampton who is investigating the relationship between patients' attitudes towards antidepressants, confidence in being able to stop them, their perception of the views of important others (their doctor, family and friends), and intentions to stop, with the actual cessation rate over six months.

ETHICAL CONSIDERATIONS

Patients with mental health problems like depression may be more sensitive than others to the demands of participation in research, but the effects of the problems in a primary care population are not so severe as to interfere with patients' capacity to understand the information provided or to give informed consent, provided patients with suicidal ideas, psychotic symptoms, and dementia are excluded. We have ensured the study aims are relevant to patients and the public through PPI input to the design, and their involvement will continue throughout to ensure that participation is voluntary, that easily understood patient information is provided, and fully informed consent obtained. Confidentiality and freedom to drop out at any time (see below) will be ensured.

In obtaining and documenting informed consent, the researcher will comply with applicable regulatory requirements and adhere to the principles of Good Clinical Practice (GCP). Discussion of objectives, risks and inconveniences of the study and the conditions under which it is to be conducted will be provided to the participant by appropriately delegated staff with knowledge in obtaining informed consent and with reference to the patient information leaflet. This information will emphasise that participation in the trial is voluntary and that the participant may withdraw from the trial at any time and for any reason. The participant will be given the opportunity to ask any questions that may arise and provided with the opportunity to discuss the study with family members, friend or an independent healthcare professional outside of the research team and time to consider the information prior to agreeing to participate.

Independent peer review through the NIHR PGfAR panel has ensured scientific quality and rigor. Ethics Committee and HRA approvals will be obtained prior to commencement of work with patients and health professionals, and any subsequent issues will be addressed with the REC or HRA offices as necessary.

PROGRAMME STEERING COMMITTEE

A Programme Steering Committee (PSC) is in place, chaired by an academic GP, and including a senior statistician, an academic psychologist, and two PPI members. The PSC will work with the Independent Data Monitoring Committee (IDMC) and be kept informed by the CI, PI, or Programme Manager. If an extension to the study is asked for, it would be the responsibility of the PSC to look in detail as to why this was needed and give an opinion which would inform the funder (NIHR) and the sponsor (University of Southampton).

INDEPENDENT DATA MONITORING COMMITTEE

In addition to the PSC, an IDMC has been set up to oversee trial conduct, chaired by an academic psychiatrist, and including an academic GP, and a senior statistician. The IDMC is tasked with looking at data, statistical and ethical aspects. Serious adverse events will be reported to the IDMC (see below) who will advise about continuation and whether interim analyses are needed. If there is a need to ask for an interim analysis of findings the IDMC will look at them but it would be the PSC who would make the ultimate decision about whether the trial might be stopped, extended or have a substantial change to its protocol.

SAFETY OF PARTICIPANTS IN THE TRIAL

We have been advised by the Medicines and Healthcare Products Regulatory Authority (MHRA) that the study is not a clinical trial of an investigational medicinal product (CTIMP), and so a Clinical Trials Authorisation (CTA) is not required. Therefore the study team will not be bound by the MHRA CTIMP regulations on alerting the sponsor and ethics committee within specific timescales of any adverse events, or potential adverse effects of the intervention or trial procedures, which are reported by patients or practitioners participating in the trial.

However, the safety of patients in the trial remains our paramount consideration and the trial coordinator will ensure that any adverse events reported by patients or practitioners will be brought to the attention of the Programme Manager at REDUCE@soton.ac.uk (Tel: 02380 591754) as quickly as possible. It will then be for immediate discussion with the Chief Investigator (CI) or in the absence of the CI, one of the Principal Investigators (PIs). The CI or PI will

decide whether or not to inform the Sponsor, the Ethics Committee, PSC and/or IDMC. The report will include the event, when the information was reported, assessment of seriousness and likely relationship to participation in the trial.

All serious adverse events (SAEs) will be reported to the Programme Manager at REDUCE@soton.ac.uk (Tel: 02380 591754) and sponsor- The University of Southampton within 24 hours of the local site becoming aware of the event. We will use the Southampton CTU's SAE Non-CTIMP Form, which asks for the nature of the event, date of onset, severity, corrective therapies given, outcome, causality (i.e. unrelated, unlikely, possible, probably, definitely) and expectedness. The Chief Investigator will assign the causality and expectedness of the event and the term should be in accordance with the latest version of MedDRA and grades given in accordance with the NCI CTCAE v4.03. Additional information will be provided as soon as possible if the event has not resolved at the time of reporting.

The Chief Investigator or Programme Manager will notify the REC of related and unexpected SAEs occurring during the study according to the following timelines; fatal and life-threatening within 7 days of notification and non-life threatening within 15 days.

WITHDRAWAL FROM THE STUDY

Patient and practitioner participants will be free to withdraw consent at any time without providing a reason. Notice of withdrawal should be given to the Programme Manager at Southampton, via email to REDUCE@soton.ac.uk. When withdrawn, patient participants will continue to receive standard clinical care from their practitioner. Follow up data will continue to be collected (unless the participant has specifically stated that they do not want this to happen).

CONFIDENTIALITY

The research team will preserve the confidentiality of participants taking part in the study. The investigators will ensure that participant's anonymity will be maintained and that their identities are protected from unauthorised parties. On trial documents and files participants will not be identified by their names, but by an identification code. The key to identification codes will be kept in a separate room to the trial data documents and files, in a locked cabinet within the University of Southampton Primary Care department at Aldermoor Health Centre.

INDEMNITY

The University of Southampton's public and professional indemnity insurance policy provides an indemnity to UoS employees for their potential liability for harm to participants during the conduct of the research. This does not in any way affect an NHS' Trust's or GP Practice's responsibility for any clinical negligence on the part of its staff.

DATA HANDLING

Participant data will be entered on laptop computers on site and retained at the University of Southampton in accordance with the General Data Protection Regulation (2018). The CI will be responsible for ensuring the accuracy, completeness, and timeliness of the data entered. Participant data will be pseudo-anonymised by assigning each participant a participant identifier code which will be used to identify the participant during the study and for any participant- specific clarification between the University and participating practices.

The Informed Consent Form will specify the participant data to be collected and how it will be managed or might be shared; including handling of all Patient Identifiable Data (PID) and sensitive PID adhering to relevant data protection law. Trained personnel with specific roles assigned will be granted access to the electronic patient data.

MONITORING

Data stored at the Universities of Southampton, Liverpool, and Hull will be checked for missing or unusual values (range checks) and checked for consistency within participants over time. Any suspect data will be returned to the researcher or practice in the form of data queries.

The participants' anonymised data may also be reviewed by the Independent Data Monitoring Committee. Details will remain confidential and participants' names will not be recorded outside the University.

RECORD RETENTION AND ARCHIVING

Study documents will be retained in a secure location during and after the trial has finished, in accordance with the sponsor University of Southampton's regulations. After study closure the CI will maintain all source documents and study related documents and retain them for a period of 10 years.

PUBLICATION AND DATA SHARING POLICY

A REDUCE programme dissemination group has been established, whose purpose is to oversee the planned outputs from the REDUCE programme, and agree on data sharing arrangements. This group comprise the CI Tony Kendrick (TK) in Southampton and one PI from each centre (York (Simon Gilbody), Liverpool (Chris Dowrick), and London (Glyn Lewis). All correspondence will be handled in the first instance by the Programme Manager Wendy O'Brien on behalf of TK. A copy of the publication and data sharing policy is available on request.

REFERENCES

Band R, James E, Culliford D, Dimitrov B, Kennedy A, Rogers A, Vassilev I. Development of a measure of collective efficacy within personal networks: A complement to self-efficacy in self-management support? Patient Education and Counselling 2019; 102: 1389–96. doi:10.1016/j.pec.2019.02.026

Borm GF, Fransen J, Lemmens WA. A simple sample size formula for analysis of covariance in randomized clinical trials. J Clin Epidemiol. 2007 Dec;60(12):1234-8. doi: 10.1016/j.jclinepi.2007.02.006.

Brazier JE, Roberts J (2004). The estimation of a preference-based measure of health from the SF-12. Medical Care 42, 851–859.

Brazier J, Connell J, Papaioannou D, Mukuria C, Mulhern B, Peasgood T, Lloyd Jones M, Paisley S, O'Cathain A, Barkham M, Knapp M, Byford S, Gilbody S, Parry G (2014). A systematic review, psychometric analysis and qualitative assessment of generic preference-based measures of health in mental health populations and the estimation of mapping functions from widely used specific measures. Health Technology Assessment 18(34) DOI: http://dx.doi.org/10.3310/hta18340.

Charlesworth G, Burnell K, Hoe J, Orrell M, Russell I. Acceptance checklist for clinical effectiveness pilot trials: a systematic approach. BMC Medical Research Methodology 2013;13:78. https://doi.org/10.1186/1471-2288-13-78

Clarke DJ, Hawkins R, Sadler E, Harding G, McKevitt C, et al. (2014) Introducing structured caregiver training in stroke care: findings from the TRACS process evaluation study. BMJ Open 4:e004473 doi:10.1136/bmjopen-2013-004473.

Clayton AH, McGarvey EL, Clavet GJ (1997). The Changes in Sexual Functioning Questionnaire (CSFQ): development, reliability, and validity. Psychopharmacology Bulletin 33(4):731-45.

Coupland C, Dhiman P, Morriss R, Arthur A, Barton G, Hippisley-Cox J (2011). Antidepressant use and risk of adverse outcomes in older people: population based cohort study. BMJ 343:d4551 doi:10.1136/bmj.d4551.

Cruickshank G, MacGillivray S, Bruce D, Mather A, Matthews K, Williams B (2008) Cross-sectional survey of patients in receipt of long-term repeat prescriptions for antidepressant drugs in primary care. Mental Health in Family Medicine 5, 105–9.

Damschroder LJ, Aron DC, Keith RE, Kirsh SR, Alexander JA, et al. (2009) Fostering implementation of health services research findings into practice: a consolidated framework for advancing implementation science. Implementation Science 4:50 doi:10.1186/1748-5908-4-50.

Devlin N, Shah K, Feng Y, Mulhern B, van Hout B. Valuing health-related quality of life: an EQ-5D-5L value set for England. Office of Health Economics: London, UK; 2016.

Drew P, Chatwin J, Collins S. (2000). Conversation Analysis: a Method for Research into Interactions between Patients and Health-care Professionals. Health Expectations, Vol. 4, pp. 58-70.

Eldridge SM, Ashby D, Kerry S (2006). Sample size for cluster randomized trials: effect of coefficient of variation of cluster size and analysis method. International Journal of Epidemiology 35, 1292–1300.

Eldridge SM, Lancaster GA, Campbell MJ, Thabane L, Hopewell S, Coleman CL, Bond CM. Defining Feasibility and Pilot Studies in Preparation for Randomised Controlled Trials: Development of a Conceptual Framework. PLOS ONE 2016;11(3):e0150205 doi: https://doi.org/10.1371/journal.pone.0150205.

Eveleigh R, Grutters J, Muskensa E, Oude Voshaard R, van Weel C, Speckens A, Lucassen P (2014) Cost-utility analysis of a treatment advice to discontinue inappropriate long-term antidepressant use in primary care. Family Practice 31, 5, 578-584.

Eveleigh RH (2015). Inappropriate long-term antidepressant use in primary care: a challenge to change (PhD thesis). Radboud University, Nijmegen.

Fava GA, Gatti A, Belaise C, Guidi J, Offidani E (2015). Withdrawal Symptoms after Selective Serotonin Reuptake Inhibitor Discontinuation: A Systematic Review. Psychotherapy and Psychosomatics 84, 72-81 (DOI:10.1159/000370338)

Finch TL, Bamford C, Deary V, Sabin N, Parry SW (2014) Making sense of a cognitive behavioural therapy intervention for fear of falling: qualitative study of intervention development. BMC Health Services Research 14:436. doi: 10.1186/1472-6963-14-436.

Gilbody S, Richards D, Barkham M (2007) Diagnosing depression in primary care using self-completed instruments: UK validation of PHQ-9 and CORE-OM. British Journal of General Practice 57, 650–652.

Goodwin GM, Price J, De Bodinat C, Laredo J. Emotional blunting with antidepressant treatments: A survey among depressed patients. J Affect Disorders 2017;221:31-35. doi: 10.1016/j.jad.2017.05.048.

Group EQ (1990). EuroQol - a new facility for the measurement of health-related quality of life. Health Policy 16, 199–208.

Hooker L, Small R, Humphreys C, Hegarty K, Taft A (2015) Applying normalization process theory to understand implementation of a family violence screening and care model in maternal and child health nursing practice: a mixed method process evaluation of a randomised controlled trial. Implement Science 10: 39. doi: 10.1186/s13012-015-0230-4.

Howie JG, Heaney DJ, Maxwell M, Walker JJ (1998). A comparison of a Patient Enablement Instrument (PEI) against two established satisfaction scales as an outcome measure of primary care consultation. Family Practice 15:165–71.

Ilyas S, Moncrieff J. Trends in prescriptions and costs of drugs for mental disorders in England, 1998-2010. Br J Psychiatry 2012;200:393-8.

Independent Research Service of the House of Commons Library, 2008. http://researchbriefings.parliament.uk/

Johnson CF, Macdonald HJ, Atkinson P, Buchanan AI, Downes N, Dougall N (2012) Reviewing long-term antidepressants can reduce drug burden: a prospective observational cohort study. British Journal of General Practice 62 (11), e773-e779. (doi: 10.3399/bjgp12X658304).

Keller A, McGarvey EL, Clayton AH (2006). Reliability and construct validity of the Changes in Sexual Functioning Questionnaire short-form (CSFQ-14). Journal of Sexual and Marital Therapy 32(1):43-52.

Kendrick, T., Chatwin, J., Dowrick, C., Tylee, A., Morriss, R., Peveler, R., Leese, M., McCrone, P., Harris, T., Moore, M., Byng, R., Brown, G., Barthel, S., Mander, H., Ring, A., Kelly, V., Wallace, V., Gabbay, M., Craig, T. and Mann, A. (2009) Randomised controlled trial to determine the clinical effectiveness and cost-effectiveness of selective serotonin reuptake inhibitors plus supportive care, versus supportive care alone, for mild to moderate depression with somatic symptoms in primary care: the THREAD (THREshold for AntiDepressant response) study. Health Technology Assessment, 13, (22), i-182. (doi:10.3310/hta13220).

Kendrick T, Stuart B, Newell C, Geraghty AWA, Moore M (2015a). Changes in rates of recorded depression in English primary care 2003-2013: time trend analyses of effects of the economic recession, and the GP contract quality outcomes framework (QOF). Journal of Affective Disorders, 180, 68-78. (doi:10.1016/j.jad.2015.03.040).

Kendrick T, Stuart B, Newell C, Geraghty AWA, Moore M (2015b). Did NICE guidelines and the Quality Outcomes Framework change GP antidepressant prescribing in England? Observational study with time trend analyses 2003-2013. Journal of Affective Disorders, 186, 171-177. (doi:10.1016/j.jad.2015.06.052).

Kennedy A, Chew-Graham C, Blakeman T, Bowen A, Gardner C, et al. (2010) Delivering the WISE (Whole Systems Informing Self-Management Engagement) training package in primary care: learning from formative evaluation. Implement Science 5: 7. doi:10.1186/1748-5908-5-7.

Kroenke K, Spitzer RL, Williams JB (2001). The PHQ-9: validity of a brief depression measure. Journal of General and Internal Medicine 16, 606-613.

Kroenke K, Spitzer RL, Williams JBW, Monahan PO, Löwe B (2007). Anxiety disorders in primary care: prevalence, impairment, comorbidity, and detection. Annals of Internal Medicine 146:317-325.

Lamers LM, Bouwmans CA, van Straten A, Donker MC, Hakkaart L (2006). Comparison of EQ-5D and SF-6D utilities in mental health. Health Economics 15, 1229–36.

Little P, Lewith G, Webley F, Evans M, Beattie A, Middleton K, Barnett J, Ballard K, Oxford F, Smith P, Yardley L, Hollinghurst S, Sharp D (2008) Randomised controlled trial of Alexander Technique for chronic and recurrent back pain. British Medical Journal, 42, (12), 965-968. (doi:10.1136/bmj.a884). (PMID:19096019).

Maheswaran, H., Weich, S., Powell, J., & Stewart-Brown, S (2012). Evaluating the responsiveness of the Warwick Edinburgh Mental Well-Being Scale (WEMWBS): Group and individual level analysis. Health and Quality of Life Outcomes, 10 (1):156. http://www.hqlo.com/content/10/1/156

Mann, R., Gilbody, S., & Richards, D. (2009). Putting the 'Q' in depression QALYs: a comparison of utility measurement using EQ-5D and SF-6D health related quality of life measures. Social psychiatry and psychiatric epidemiology, 44(7), 569-578. doi:10.1007/s00127-008-0463-5

May CR, Mair F, Finch T, MacFarlane A, Dowrick C, et al. (2009) Development of a theory of implementation and integration: Normalization Process Theory. Implementation Science 4: 29.

Meakin R, Weinman J (2002). The 'Medical Interview Satisfaction Scale' (MISS-21) adapted for British general practice. Family Practice 19(3):257-63.

Michie S, Van Stralen M, West R (2011). The behaviour change wheel: a new method for characterising and designing behaviour change interventions. Implementation Science 6:42 doi:10.1186/1748-5908-6-42.

Middleton DJ, Cameron IM, Reid IC. Continuity and monitoring of antidepressant therapy in a primary care setting. Quality in Primary Care 2011; 19(2): 109–113.

Moore M, Yuen HM, Dunn N, Mullee MA, Maskell J, Kendrick T. Explaining the rise in antidepressant prescribing: a descriptive study using the general practice research database. BMJ 2009;339:b3999.

Mulhearn B, Mukuria C, Barkham M, Knapp M, Byford S, Soeteman D, Brazier J (2014). Using generic preference-based measures in mental health: psychometric validity of the EQ-5D and SF-6D. British Journal of Psychiatry 205, 236-243.

Murray E, Treweek S, Pope C, MacFarlane A, Ballini L, et al. (2010) Normalisation process theory: a framework for developing, evaluating and implementing complex interventions. BMC Med 8: 63.

NICE (2009) Depression in adults: The treatment and management of depression in adults. NICE, London.
Peasgood T, Brazier J, Papaioannou D (2012). A systematic review of the validity and responsiveness of EQ-5D and SF-6D for depression and anxiety. HEDS Discussion Paper No.12.15 Health Economics and Decision Science, School of Health and Related Research, University of Sheffield.

Peasgood T, Brazier J, Papaioannou D (2012). A systematic review of the validity and responsiveness of EQ-5D and SF-6D for depression and anxiety. HEDS Discussion Paper No.12.15 Health Economics and Decision Science, School of Health and Related Research, University of Sheffield.

Rosenbaum, J.F., Fava, M., Hoog, S.L. et al (1988). Selective serotonin reuptake inhibitor discontinuation syndrome: a randomized clinical trial. Biological Psychiatry, 44:77-87.

Sinclair JE, Aucott LS, Lawton K, Reid IC, Cameron IM. The monitoring of longer term prescriptions of antidepressants: Observational study in a primary care setting. Fam Pract 2014; 31: doi:/10.1093/fampra/cmu019.

Spitzer RL, Kroenke K, Williams JB, et al; A brief measure for assessing generalized anxiety disorder: the GAD-7. Archives of Internal Medicine 2006;166:1092-7.

Stewart-Brown, S. L., Platt, S., Tennant, A., Maheswaran, H., Parkinson, J., Weich, S., Clarke, A. (2011). The Warwick-Edinburgh Mental Well-being Scale (WEMWBS): a valid and reliable tool for measuring mental well-being in diverse populations and projects. Journal of Epidemiology and Community Health, 65 (Suppl 2), A38-A39.

Thomas LH, French B, Burton CR, Sutton C, Forshaw D, et al. (2014) Evaluating a systematic voiding programme for patients with urinary incontinence after stroke in secondary care using soft systems analysis and Normalisation Process Theory: findings from the ICONS case study phase. International Journal of Nursing Studies 51: 1308-1320.

Uher R, Farmer A, Henigsberg N, et al (2009). Adverse reactions to antidepressants. British Journal of Psychiatry 195:202-210. DOI: 10.1192/bjp.bp.108.061960.

Ware JE, Kosinski M, Keller SD (1996). A 12-Item Short-Form Health Survey: Construction of scales and preliminary tests of reliability and validity. Medical Care, 34 (3), 220–233.

Southampton Clinical Trials Unit

Reviewing long-term antidepressant use by careful monitoring in everyday practice (REDUCE)

Statistical Analysis Plan

Trial registration number:	https://www.isrctn.com/ISRCTN12417565 Registered 07/10/2019
Protocol title and version number:	https://trialsjournal.biomedcentral.com/articles/10.1186/s13063-020-04338-7
SAP version number:	1
SAP date:	21 April 2022

To be approved and reviewed by:

	Name	Signature	Date
**Chief Investigator**	Professor Tony Kendrick	[signature]	21/4/2022
**Trial Statistician (author)**	Dr Beth Stuart	[signature]	21/4/2022

UKCRC Registered Clinical Trials Units

NIHR | National Institute for Health Research

Table of Contents

Reviewing long-term antidepressant use by careful monitoring in everyday practice (REDUCE) 1

1 Introduction 4

1.1 Purpose of SAP 4

1.2 Trial background and rationale (from ISRCTN registration) 4

1.3 Objectives (from published protocol[1]) 4

1.4 Definition of endpoints 4

1.5 Analysis principles 5

2 Design considerations 6

2.1 Description of trial design 6

2.2 Trial power and sample size 6

2.3 Randomisation details 6

2.4 Timing of planned analyses 6

3 Statistical considerations 7

3.1 Definition of analysis populations 7

3.2 Analysis software 7

3.3 Methods for handling data 7

3.4 Definition of key derived variables 8

3.5 General principles for reporting and analysis 9

4 Planned analyses and reporting 10

4.1 Disposition of the study population 10

4.2 Protocol deviations 10

4.3 Baseline and demographic characteristics 10

4.4 Primary endpoint 10

4.5 Secondary endpoints ... 10

4.6 Additional analyses ... 11

4.7 Safety reporting ... 11

5 References ... 12

1 Introduction

1.1 Purpose of SAP

This statistical analysis plan (SAP) describes in detail the methods that will be used to analyse the data collected as part of the REDUCE trial. This will form the basis of the final trial publication. The final analysis will follow the SAP to ensure that the analyses are conducted in a scientifically valid manner and to avoid post hoc decisions which may affect the interpretation of the statistical analysis. Any deviations from the SAP will be detailed in the final report.

1.2 Trial background and rationale (from ISRCTN registration)

Work Stream 5 (WS5) of the REDUCE programme aims to determine the effectiveness of online (Internet) interventions which support practitioners and guide patients on coming off antidepressants, together with psychological practitioner telephone calls to support the patients.

We will assess the effectiveness of the interventions in terms of reductions in antidepressant use in the absence of worsening of depression, and assess patients' and practitioners' use of the interventions (automatically recorded by the Southampton 'LifeGuide' software used for the Internet guidance).

1.3 Objectives (from published protocol[1])

1. To determine the effectiveness of the intervention through a randomised controlled trial
2. To estimate cost-effectiveness from a health and personal social service perspective, with a sensitivity analysis from a societal perspective.

   The analysis required to evaluate the first of these objectives will be set out in this Statistical Analysis Plan. The analysis required to evaluate the second objective will be set out in a separate Health Economics Analysis Plan.

1.4 Definition of endpoints

1.4.1 Definition of primary endpoint

The PHQ-9 scores for depressive symptoms at 6 months.

1.4.2 Definition of secondary endpoints

- The PHQ-9 score over the full 12 month period as a repeated measure analysis
- Discontinuation of antidepressants at 6 months
- Antidepressant withdrawal symptoms measured on the Discontinuation Emergent Signs and Symptoms scale (DESS) over the trial period
- Anxiety on the GAD-7 measure of generalised anxiety disorder over the trial period
- Wellbeing scores over the trial period on the Warwick-Edinburgh Mental Wellbeing Scale (WEMWBS)
- Modified version of the Medical Informant Satisfaction Scale (MISS) over the trial period to measure patient satisfaction over the follow-up period.
- Patient enablement measured by the Patient Enablement Instrument (PEI) over the trial period

1.5 Analysis principles

All analyses will be reported in line with the CONSORT 2010 extension to cluster randomised trials[2] and non inferiority trials[3] and Southampton Clinical Trials Unit (SCTU) standard operating procedure (SOP) on planning, implementing and reporting statistical analyses (CTU/SOP/5058).

2 Design considerations

2.1 Description of trial design

This is a pragmatic multicentre cluster randomised non-inferiority trial.

2.2 Trial power and sample size

To have 90% power, with a one-sided alpha of 2.5%, to establish non-inferiority in terms of depressive symptoms within two points (estimated to be the minimal clinically important difference) on the PHQ-9 at 6 months (standard deviation 5.4), we need 155 patients followed up in each arm. Assuming a variable cluster size of between 1 and 7 per practice (mean 3) and an intra-cluster correlation coefficient of 0.012 (from the Health Technology Assessment THREAD trial of treating mild-to-moderate depression in primary care[4]), gives a 1.033 cluster design effect (based on a coefficient of variation of 1.5/3 = 0.5, using the formula of Eldridge[5]). Anticipating 20% do not comply with the intervention and/or are lost to follow-up, we need to randomise (155 × 2 × 1.036) / 0.8 = 402 patients (201 per arm) from 134 practices (67 per arm).

Following discussion with the study steering committees and the funder, the sample size was amended to 360 participants. This allows for a correlation between baseline and follow-up scores with a deflation factor of $1-p^2$. At the time of review we observed a correlation of 0.47 but were unsure whether this would persist to the end of follow up for all participants. If we assume the more conservative estimate of correlation of 0.26 (the bottom end of the CI), the target sample size to achieve 90% power would 375. We assume the final figure will be somewhere in between 0.26 and 0.47, and that a sample of around 360 will therefore provide us with the necessary 90% power to test reliably for non-inferiority of the intervention in terms of depressive symptoms.

2.3 Randomisation details

Randomisation of practices is by computerised sequence generation, and minimisation with a random element using three factors to avoid imbalance between the two arms: practice size (large/small), location (urban/rural) and social deprivation (dichotomised around the median Index of Multiple Deprivation score). The allocation ratio is 1:1 but there is a random element to the minimisation algorithm and so we might not expect perfect balance to the randomisation.

2.4 Timing of planned analyses

2.4.1 Interim analyses and early stopping

No interim analysis is planned and no pre-specified stopping rules have been established.

2.4.2 Final analysis

End of study is defined as when the last patient has had their last data collected, cleaned and verified.

3 Statistical considerations

3.1 Definition of analysis populations

3.1.1 Intention-to-treat analysis population

This population includes all randomised practices and all patients recruited within them regardless of treatment compliance. This includes the participants recruited during WP4 and treated as an internal pilot. All summaries and analysis will be on the modified ITT population unless otherwise specified, i.e. the population as randomised but without missing data imputed. The ITT analysis will be the primary analysis.

3.1.2 Per-protocol analysis population

In a non-inferiority trial where some patients do not comply with treatment as randomised, the difference between the arms can appear reduced and the groups look more similar, leading to the incorrect conclusion of non-inferiority. A per-protocol analysis would analyse individuals based on their compliance with treatment as randomised, excluding non-compliant participants, which may give a more conservative estimate of effect for non-inferiority. However, the exclusion of some participants after randomisation can potentially lead to bias. Therefore, we will present both intention-to-treat and per-protocol analyses. The ITT analysis will be the primary and the per-protocol analyses will be treated as secondary.

3.1.3 CACE analysis population

We will also undertake a complier-average causal effect analysis, which is another approach for dealing with non-compliance that compares compliant participants in the intervention group with those in the control group whose characteristics are similar enough to the intervention group compliers to suggest they too would have complied with the intervention given the opportunity to do so.[6] Compliance for these analyses in the intervention arm will be defined as completing the first session of the LifeGuide programme within 6 months of recruitment (anticipating >90%). The first session will have information about antidepressant treatment, the rationale for attempting withdrawal, and how withdrawal should be attempted under supervision. We would expect patients to benefit from that session even if they do not log on again.

3.2 Analysis software

SAS v9.4 or higher, or Stata v15.1 or higher will be used for all analyses.

3.3 Methods for handling data

3.3.1 Withdrawal from trial

All data up until the point of patient withdrawal from the trial will be used in analyses unless the patient withdrew consent and does not wish for the data already collected prior to withdrawal to be used for the trial.

If a practice withdraws from the trial, no further patients will be recruited. All data on patients collected until that point will be used and any patients recruited will continue to be followed up in accordance with the trial schedule.

3.3.2 Missing data

The primary analysis will be of complete cases.

If more than 2 items in the PHQ-9 and the GAD7 have missing values, the total score will be missing. If one or two items are missing, the score will be imputed with the mean of the non-missing scores before summing.

We will examine the structure and pattern of missing data and, if appropriate, will present a sensitivity analysis based on data imputed using a chained equations multiple imputation model. The imputation model would include the outcome measure, baseline value of the outcome, randomisation group, clustering by practice and all covariates included in the analysis model (see below)

3.3.3 Outliers

No methods will be used to handle outliers in the data, except in the regression models. If outliers are found then firstly the source data will be checked. If the source data is correct, then a sensitivity analysis will be performed excluding them from the analysis.

3.3.4 Assumption checking and alternative methods

Assumptions for linear regression models (linearity, normality, homoscedasticity) will be checked using scatter plots of standardized residuals against fitted values, and qq plots. If linear models are not appropriate a log-linear transformation will be used.

3.4 Definition of key derived variables

The PHQ-9[7] is a self-complete questionnaire taking approximately 3 min to complete. Each item is scored 0-3 with a total possible score ranging from 0-27. Higher scores indicate more severe symptoms.

The Generalised Anxiety Disorder Assessment 7-item version[8] is a self-report measure of anxiety symptoms. Each item is scored from 0-3 with a total possible score ranging from 0-21. Higher scores indicate more severe symptoms.

Discontinuation of antidepressants is deemed to have occurred once the patient has stopped taking them for 2 months. This is a binary outcome (did not discontinue/did discontinue).

The Warwick–Edinburgh Mental Wellbeing Scale (WEMWBS)[9] measures both subjective experiences of happiness and life satisfaction (the 'hedonic perspective'), and positive psychological functioning, good relationships with others and self-realisation (the 'eudaimonic perspective'). Each item is answered on a 1 to 5 Likert scale and summed to give a total score ranging form 14 to 70. Higher scores indicate greater wellbeing.

Antidepressant withdrawal symptoms are measured using the Discontinuation Emergent Signs and Symptoms scale[10], a brief self-report measure on which participants can indicate the presence of, and

changes in, 43 possible antidepressant withdrawal symptoms. The total number of symptoms is summed.

The 29-item ‘Medical Interview Satisfaction Scale’ (MISS-29) was developed in the USA to assess patient satisfaction with individual doctor-patient consultations and has been shown to be valid and reliable in UK primary care[11].

The modified Patient Enablement Instrument[12] asks 6 questions about the patients’ ability to understand their problems and cope with illness. These are rated on a 7 point Likert scale (0=strongly agree to 7=strongly disagree). A mean overall score can be calculated, with lower scores equating to better enablement.

3.5 General principles for reporting and analysis

The following general principles for reporting and analysis will be used:

- 5% two-sided level of statistical significance, with corresponding 95% confidence intervals presented where applicable.
- No adjustments for multiplicity are planned.
- Summary statistics will include either mean, standard deviation, and/or median, interquartile range.
- Treatment groups will be labelled in the tables as Intervention Group and Control Group accordingly, and a total column will be included in tables were applicable.

4 Planned analyses and reporting

4.1 Disposition of the study population

A CONSORT diagram (see Appendix 1) will be produced showing a clear account of all practices and patients who entered the trial- see below for an example figure. Withdrawal information including the primary reasons of discontinuation will be summarised and presented by where this is known.

4.2 Protocol deviations

A listing of all Major or Potential/Serious Breach (Major protocol deviations with potential to affect patient safety/data) and Potential/Serious Breach (with actual affect to patient safety/data, Major/Potential/Serious Breach of GCP guidelines or consistent non-compliance by site) will be produced (by patient and site where applicable)

4.3 Baseline and demographic characteristics

Summary statistics will be produced and presented by group for demographic and baseline characteristics but no comparisons will be undertaken, rather the clinical importance of any imbalance will be noted. If there are imbalances of clinical importance in variables not listed in 4.4 below, we will control for these as covariates in the analyses.

4.4 Primary endpoint

The primary outcome, that is, the differences at 6 months between intervention and control in depression as measured by the PHQ-9, will be analysed using a linear mixed model, adjusting for duration of depression, past history of depression, age, gender, marital status, no. of dependents, ethnic group, education level, economic position (employment status), and accommodation status, baseline depression score, anxiety score, internet use and clustering, including practice as a random effect. These covariates have been chosen based on their known relationship with the outcome from previous literature.

The model will use all the observed data and makes the assumption that missing PHQ-9 scores are missing completely at random. We will examine the lower limit of the 95% confidence interval to establish non-inferiority.

We will then undertake the same analyses using the per protocol population and the CACE population.

Whilst the ITT analysis will be the primary analysis, we will report the analyses based on all three approaches and interpret the findings cautiously in light of any differences between approaches that may emerge. This analysis will form the core of the publication of the trial results.

4.5 Secondary endpoints

The discontinuation of antidepressants will be evaluated at 6 months as a binary outcome in a mixed logistic regression model, using the same modelling approach and covariates as for the primary outcome.

Analysis of all other secondary outcomes will also be conducted using generalised linear mixed regression models adjusting for stratification variables, the potential confounders listed in 4.4 above and the baseline value of the outcome. These will be evaluated as repeated measures so all models will allow for the clustering of observations within participants over time and of participants within practices.

4.6 Additional analyses

No subgroup analyses are planned. Any post-hoc analyses will be exploratory only. Health economic analyses will be undertaken and a separate Health Economics Analysis Plan will be prepared.

In accordance with the CONSORT recommendations for cluster randomised trials, we will also report the ICC for the primary outcome.

Due to the COVID-19 pandemic and subsequent lockdown period, it is possible that there may be changes to the key outcomes unrelated to randomisation group. We will therefore look at the scores in each arm in the pre-, peri- and post-COVID periods in the whole study population. We will use descriptive statistics and graphical representations to explore any trends and aim to control for any time varying effect on outcomes in a sensitivity analysis

4.7 Safety reporting

A listing of serious adverse events (SAEs) will be provided for all related/unrelated SAEs. If required, a summary table will also be presented.

5 References

1. Kendrick, T. *et al.* REDUCE (Reviewing long-term antidepressant use by careful monitoring in everyday practice) internet and telephone support to people coming off long-term antidepressants: Protocol for a randomised controlled trial. *Trials* **21**, 1–15 (2020).

2. Campbell, M. K., Piaggio, G., Elbourne, D. R. & Altman, D. G. Consort 2010 statement: Extension to cluster randomised trials. *BMJ* **345**, (2012).

3. Piaggio, G., Elbourne, D. R., Pocock, S. J., Evans, S. J. W. & Altman, D. G. Reporting of Noninferiority and Equivalence Randomized Trials: Extension of the CONSORT 2010 Statement. *JAMA* **308**, 2594–2604 (2012).

4. Kendrick, T. *et al.* Randomised controlled trial to determine the clinical effectiveness and cost-effectiveness of selective serotonin reuptake inhibitors plus supportive care, versus supportive care alone, for mild to moderate depression with somatic symptoms in primary care: the THREAD (THREshold for AntiDepressant response) study. *Health Technol. Assess.* **13**, iii–iv, ix (2009).

5. Eldridge, S. M., Ashby, D. & Kerry, S. Sample size for cluster randomized trials: effect of coefficient of variation of cluster size and analysis method. *Int. J. Epidemiol.* **35**, 1292–1300 (2006).

6. Angrist, J. D., Imbens, G. W. & Rubin, D. B. Identification of Causal Effects Using Instrumental Variables. *J. Am. Stat. Assoc.* **91**, 444–455 (1996).

7. Kroenke, K., Spitzer, R. L., Williams, J. B. W., Monahan, P. O. & Löwe, B. Anxiety disorders in primary care: Prevalence, impairment, comorbidity, and detection. *Ann. Intern. Med.* **146**, 317–325 (2007).

8. Spitzer, R. L., Kroenke, K., Williams, J. B. W. & Löwe, B. A brief measure for assessing generalized anxiety disorder: The GAD-7. *Arch. Intern. Med.* **166**, 1092–1097 (2006).

9. Stewart-Brown, S. *et al.* The Warwick-Edinburgh Mental Well-being Scale (WEMWBS): a valid and reliable tool for measuring mental well-being in diverse populations and projects. *J Epidemiol Community Heal.* **65**, A38–A39 (2011).

10. Rosenbaum, J. F., Fava, M., Hoog, S. L., Ascroft, R. C. & Krebs, W. B. Selective serotonin reuptake inhibitor discontinuation syndrome: a randomized clinical trial. *Biol. Psychiatry* **44**, 77–87 (1998).

11. Meakin, R. & Weinman, J. The 'Medical Interview Satisfaction Scale' (MISS-21) adapted for British general practice. *Fam. Pract.* **19**, 257–263 (2002).

12. Randomised controlled trial of Alexander technique lessons, exercise, and massage (ATEAM) for chronic and recurrent back pain. doi:10.1136/bmj.a884.

Appendix 1

REDUCE RCT Consort diagram

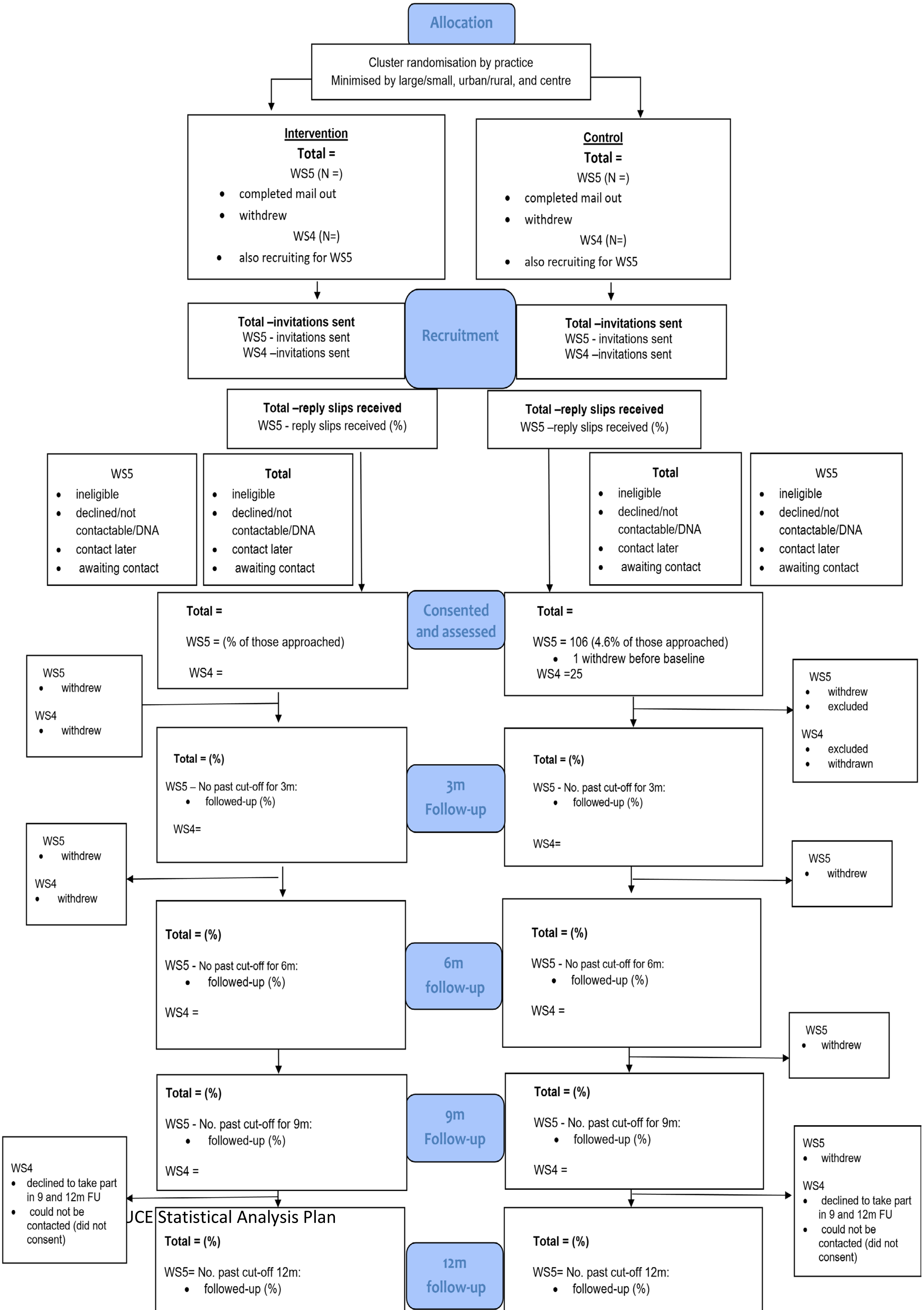
Allocation
Cluster randomisation by practice
Minimised by large/small, urban/rural, and centre
Intervention
Total =
WS5 (N =)
• completed mail out
• withdrew
WS4 (N=)
• also recruiting for WS5
Control
Total =
WS5 (N =)
• completed mail out
• withdrew
WS4 (N=)
• also recruiting for WS5
Recruitment
Total –invitations sent
WS5 - invitations sent
WS4 –invitations sent
Total –invitations sent
WS5 - invitations sent
WS4 –invitations sent
Total –reply slips received
WS5 - reply slips received (%)
Total –reply slips received
WS5 –reply slips received (%)
WS5
• ineligible
• declined/not contactable/DNA
• contact later
• awaiting contact
Total
• ineligible
• declined/not contactable/DNA
• contact later
• awaiting contact
Total
• ineligible
• declined/not contactable/DNA
• contact later
• awaiting contact
WS5
• ineligible
• declined/not contactable/DNA
• contact later
• awaiting contact
Consented and assessed
Total =
WS5 = (% of those approached)
WS4 =
Total =
WS5 = 106 (4.6% of those approached)
• 1 withdrew before baseline
WS4 =25
WS5
• withdrew
WS4
• withdrew
WS5
• withdrew
• excluded
WS4
• excluded
• withdrawn
3m Follow-up
Total = (%)
WS5 – No past cut-off for 3m:
• followed-up (%)
WS4=
Total = (%)
WS5 - No. past cut-off for 3m:
• followed-up (%)
WS4=
WS5
• withdrew
WS4
• withdrew
WS5
• withdrew
6m follow-up
Total = (%)
WS5 - No past cut-off for 6m:
• followed-up (%)
WS4 =
Total = (%)
WS5 - No past cut-off for 6m:
• followed-up (%)
WS4 =
WS5
• withdrew
9m Follow-up
Total = (%)
WS5 - No. past cut-off for 9m:
• followed-up (%)
WS4 =
Total = (%)
WS5 - No. past cut-off for 9m:
• followed-up (%)
WS4 =
WS4
• declined to take part in 9 and 12m FU
• could not be contacted (did not consent)
WS5
• withdrew
WS4
• declined to take part in 9 and 12m FU
• could not be contacted (did not consent)
12m follow-up
Total = (%)
WS5= No. past cut-off 12m:
• followed-up (%)
Total = (%)
WS5= No. past cut-off 12m:
• followed-up (%)
