# Supplementary material for: Internet and Telephone Support for Discontinuing Long-Term Antidepressants: The REDUCE Cluster Randomized Trial
Source: JAMA Netw Open. 2024 Jun 24;7(6):e2418383. doi: 10.1001/jamanetworkopen.2024.18383 (PMC11197448; doi:10.1001/jamanetworkopen.2024.18383)
Supplement: Supplement 2. — eAppendix 1. The Patient Online Intervention “ADvisor” eReferences 1. eAppendix 2. The Practitioner Online Intervention “AdvisorHP” eReferences 2. eAppendix 3. PWP Telephone Support [file jamanetwopen-e2418383-s002.pdf]

Supplementary Online Content

Kendrick T, Stuart B, Bowers H, et al. Internet and telephone support for discontinuing long-term antidepressants: the REDUCE cluster randomized trial. *JAMA Netw Open.* 2024;7(6):e2418383. doi:10.1001/jamanetworkopen.2024.18383

eAppendix 1. The Patient Online Intervention "*ADvisor*"
eReferences 1.
eAppendix 2. The Practitioner Online Intervention "*AdvisorHP*"
eReferences 2.
eAppendix 3. PWP Telephone Support

This supplementary material has been provided by the authors to give readers additional information about their work.

© 2024 Kendrick T et al. *JAMA Network Open.*

eAppendix 1. The Patient Online Intervention "*ADvisor*"

ADvisor is an internet-supported CBT-based intervention for patients to support their withdrawal from antidepressant treatment. It was designed through a cooperative process with patients, taking their views into account throughout its development and implementation, iteratively. The intervention development work with patients was published in 2020.[1]

The *Person-Based Approach (PBA)* was used to develop the intervention. This is a mixed methods approach developed by Lucy Yardley at the University of Southampton, to guide development of behavioural interventions.[2] It was used to integrate the results of initial in-depth open-ended qualitative interviews with patients, together with theoretical models from the published literature, to develop a prototype intervention, which was then optimised through further *'think-aloud'* interviews with patients, conducted while they tried out the prototype, and spoke out loud their thoughts on possible problems and improvements.

Guiding principles for the intervention were developed based on the important barriers and facilitators to antidepressant discontinuation, identified from the literature and our initial qualitative interviews. Necessary components were identified through consideration of the factors likely to affect target behaviours, drawing on the behaviour change wheel (BCW) and capability, opportunity, motivation-behaviour (COM-B) model of behaviour of Michie and colleagues.[3]

A *logic model* representing likely theories of change was then developed, outlining the problems, facilitators, barriers, and possible mechanisms of ingredients, and how they might influence the target outcome of antidepressant discontinuation.[4]

The intervention components developed included:

1. Information about discontinuation (how antidepressants work, withdrawal symptoms and how to minimise them, understanding the role of thoughts in mood changes, stories of others who had stopped antidepressants, and reflections on motivations to stop).
2. Cognitive and meta-cognitive techniques (self-compassion exercises, accepting thought and emotions, mindfulness exercises, cognitive de-fusion, self-monitoring of warning signs and triggers for relapse, and self-management in the face of relapse).
3. Behavioral techniques (stress-management, behavioural activation, monitoring thoughts, reflecting on values, goal-setting with new values).
4. Environment (information for family and friends, appointments with GP/NP, telephone contacts to address concerns, and engagement with online content).

The eight constituent modules developed were:

1. *How ADvisor could help patients to reduce and stop antidepressants*
2. *Reducing and stopping - encouraging patients to consider their motives*
3. *Thinking about antidepressants (and why lifelong treatment may not be necessary)*
4. *Dealing with withdrawal symptoms*
5. *Worries about stopping including withdrawal and relapse*
6. *Keeping well and looking out for warning signs of relapse*
7. *Thinking about what patients valued in life*
8. *Moving forward and how to deal with difficult feelings*

© 2024 Kendrick T et al. *JAMA Network Open.*

The intervention was menu-driven, and when patients first logged on, they could only access the Introductory module “How ADvisor can help”. After that they could access the other modules in any order according to their preference, and on each page of each module there were direct links back to the opening page, so that patients did not have to complete a whole module before they could go back to the opening page if they wished.

Screenshot of opening page of ADvisor

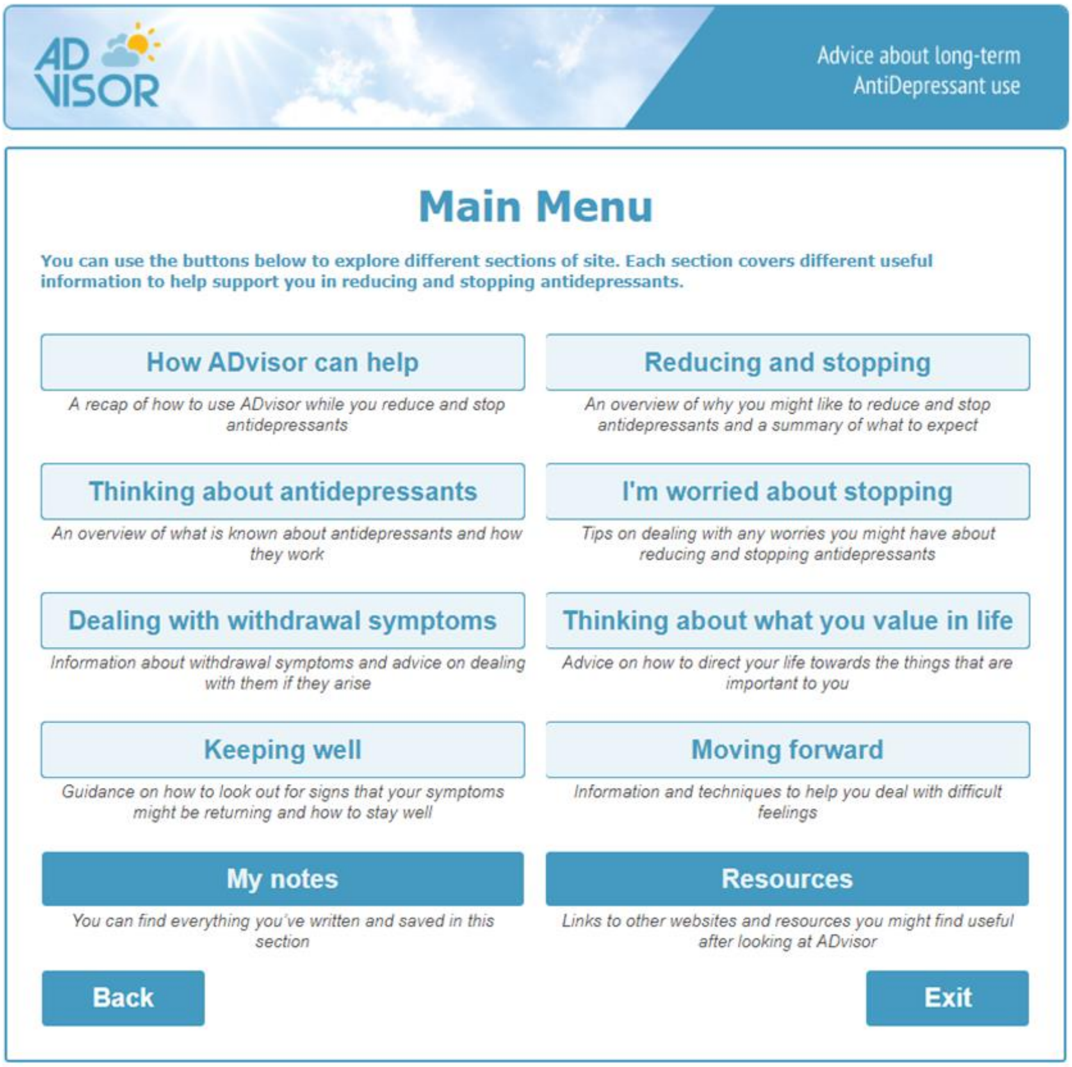

Reproduced from Kendrick T. Strategies to reduce use of antidepressants. British Journal of Clinical Pharmacology 2020 https://doi.org/10.1111/bcp.14475, with permission from John Wiley & Sons on behalf of British Pharmacological Society.

© 2024 Kendrick T et al. *JAMA Network Open.*

Intervention arm participants' use of the ADvisor online intervention

Intervention arm patients' online accessing of the modules was automatically recorded to estimate compliance and its relation to outcomes. The table below shows compliance (defined as accessing the Introductory module) was 66.3%. Of the 178 who accessed the introductory module, 9 did not log on again to access any further modules, so the denominator for the other modules was 169.

Modules of the *ADvisor* online intervention	Number who accessed the module (%)	Mean PHQ-9 at 6 months for those who accessed the module (SD)	Mean PHQ-9 at 6 months for those who did not access module (SD)	Proportion who discontinued ADs among those who accessed the module (%)	Proportion who discontinued ADs among those who did not access module (%)
Introduction to *ADvisor*	118/178 (66.3%)	4.0 (4.21)	4.1 (4.71)	49/106 (46.2%)	17/39 (43.6%)
Thinking about Antidepressants	40/169 (23.7%)	4.1 (3.95)	4.0 (4.28)	12/34 (35.3%)	52/106 (49.1%)
Dealing with withdrawal symptoms	72/169 (42.6%)	3.4 (3.58)	4.5 (4.60)	28/63 (44.4%)	36/77 (46.8%)
I'm worried about stopping	41/169 (24.3%)	4.4 (4.52)	3.9 (4.09)	7/35 (20.0%)	57/105 (54.3%)
Keeping Well	56/169 (33.1%)	4.3 (4.51)	3.8 (4.02)	26/52 (50.0%)	38/88 (43.2%)
Values and Goals	34/169 (20.1%)	4.8 (4.52)	3.8 (4.09)	15/31 (48.4%)	49/109 (45.0%)
Moving Forward	60/169 (35.5%)	4.4 (4.87)	3.7 (3.71)	27/55 (49.1%)	37/85 (43.5%)

PHQ-9 scores were slightly higher in those who consulted modules compared to those who did not, apart from that on *Dealing with withdrawal symptoms*, but not significantly. Antidepressant discontinuation was higher in those who accessed modules on: *Keeping well, Values and goals*, and *Moving forward*, and lower in those completing *Thinking about antidepressants, Dealing with withdrawal*, and *Worry about stopping*, but only significantly for *Worry about stopping* (OR 0.13, 95% CI 0.04, 0.41).

© 2024 Kendrick T et al. *JAMA Network Open.*

eReferences 1.

1. Bowers HM, Kendrick T, Glowacka M, et al. Supporting antidepressant continuation: the development and optimisation of a digital intervention for patients in UK primary care using a theory, evidence and person-based approach. BMJ Open 2020;10:e032312. https://doi.org/10.1136/bmjopen-2019-032312
2. Yardley, L., Morrison, L., Bradbury, K., Muller, I. The Person-Based Approach to Intervention Development: Application to Digital Health-Related Behavior Change Interventions. Journal of Medical Internet Research 2015;17:e30).
3. Michie S, Van Stralen M, West R. The behaviour change wheel: a new method for characterising and designing behaviour change interventions. Implementation Science 2011;6:42 doi:10.1186/1748-5908-6-42.
4. Moore GF, Audrey S, Barker M, Bond L, Bonell C, Hardeman W, Moore L, O'Cathain A, Tinati T, Wight D, Baird J. Process evaluation of complex interventions: Medical Research Council guidance. BMJ 2015;350:h1258. doi: 10.1136/bmj.h1258.

© 2024 Kendrick T et al. *JAMA Network Open.*

eAppendix 2. The Practitioner Online Intervention "*AdvisorHP"*

The practitioner intervention (ADvisor for Health Professionals or "*AdvisorHP"*) was developed to try to increase GP/NP self-efficacy to taper and discontinue patients' antidepressant treatment where appropriate. It was designed through a cooperative process with health professionals, taking their views into account throughout its development and implementation, iteratively. The intervention development work with health professionals was published in 2021.[1]

The Person-Based Approach (PBA) was used to develop the intervention. This is a mixed methods approach developed by Lucy Yardley at the University of Southampton, to guide development of behavioural interventions.[2] It was used to integrate the results of initial in-depth open-ended focus groups and individual qualitative interviews with professionals, together with theoretical models from the published literature, to develop a prototype intervention. The prototype was then optimised through further '*think-aloud'* interviews, conducted while the professionals tried out the prototype, and spoke out loud their thoughts on possible problems and improvements.

Guiding principles for the intervention were developed based on the important barriers and facilitators to antidepressant discontinuation, identified from the literature and our initial focus groups and individual interviews. Necessary components were identified through consideration of the factors likely to affect target behaviours, drawing on the behaviour change wheel (BCW) and capability, opportunity, motivation-behaviour (COM-B) model of behaviour of Michie and colleagues.[3]

A *logic model* representing likely theories of change was then developed, outlining the problems, facilitators, barriers, and possible mechanisms of ingredients, and how they might influence the target outcome of antidepressant discontinuation.[4]

The intervention components developed included:

1. Understanding the patient (including information about why patients may want to reduce and stop antidepressants), questioning the serotonin hypothesis and the negative impact this can have on patients' motivation, links to content of the patient intervention "*ADvisor"* and information on how they may use psychological and behavioural tools to manage symptoms).
2. Practical information (specific reduction schedules for all antidepressants except MAOIs, initiating the review, and managing the consultation with agenda-setting, clear guidance on when to restart antidepressants and when to allow the patient to self-manage, and signs of relapse and withdrawal symptoms).

The six constituent modules were:

1. *Why reduce, reasons why patients might want to.*
2. *Broaching the subject.*
3. *When to start tapering, patient readiness.*
4. *Reduction schedules for antidepressants.*
5. *Dealing with withdrawal symptoms and distinguishing them from relapse.*
6. *Dealing with relapse.*

It included examples of hyperbolic tapering schedules (slower, non-linear schedules) for people with a history of particular past difficulty in discontinuation, devised by our specialist pharmacist.

© 2024 Kendrick T et al. *JAMA Network Open.*

The intervention was menu-driven, and practitioners could access the modules in any order according to their preference. On each page of each module there were direct links back to the AdvisorHP opening page, so that patients did not have to complete a whole module before they could go back to the opening page if they wished.

Screenshot of AdvisorHP

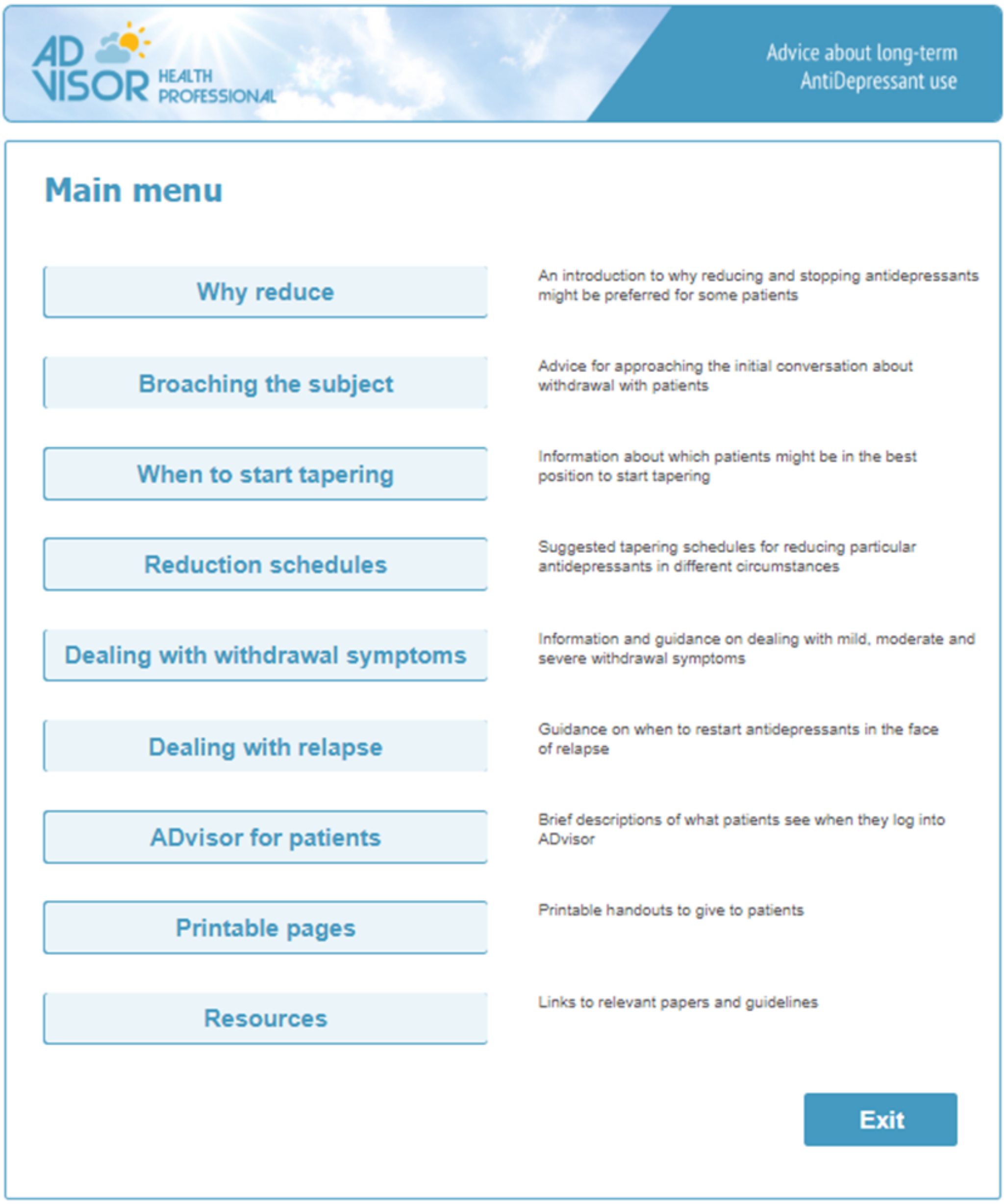

Reproduced from Kendrick T. Strategies to reduce use of antidepressants. British Journal of Clinical Pharmacology 2020 https://doi.org/10.1111/bcp.14475, with permission from John Wiley & Sons on behalf of British Pharmacological Society.

© 2024 Kendrick T et al. *JAMA Network Open.*

Example antidepressant tapering schedule from ADvisor for Health Professionals

The following example is for sertraline, the most commonly prescribed antidepressant in the UK (June 2023).

Schedule A: Suggested starting schedule for *a person who has been on it for less than two years, has no past history of distressing withdrawal, no particular fear of undergoing withdrawal over 6 weeks, and does not wish to prolong tapering*, is as follows:

Reduce every two weeks: 200mg – 150mg – 100mg – 50mg – Stop

However, reducing every four weeks (12 weeks in all) may be more practical for working patients in terms of arranging prescriptions and follow-up appointments in general practice.

Schedule B: For *those who have been on sertraline for more than two years, or are particularly anxious about withdrawal*, reducing every four weeks initially may be preferred, with follow-ups between each reduction. (by telephone or face to face if preferred).

Schedule C: For *patients who have a history of difficult or distressing withdrawal*, an even slower, hyperbolic, withdrawal schedule might be offered, as follows:

Reduce every four weeks: 200mg – 150mg – 100mg – 50mg – 25mg – 15mg – 10mg – 7.5mg – 5mg – 2.5mg – 1.25mg – Stop

Ideally this should be done using a liquid sertraline preparation arranged with the pharmacist. If a liquid preparation is not available then switching to fluoxetine liquid and tapering that preparation hyperbolically is an option. Fluoxetine 20mg is equivalent to Sertraline 50mg (and Citalopram 20mg, Escitalopram 10mg, Fluvoxamine 50mg, or Paroxetine 20mg).

A hyperbolic reduction schedule for fluoxetine liquid (4mg/ml) would be as follows (Table 1):

Table 1 Hyperbolic reduction schedule for fluoxetine liquid

Step	mg/day	ml/day
1	20	5
2	12	3
3	8	2
4	4.8	1.2
5	3.2	0.8
6	1.6	0.4
7	0.8	0.2
8	0.4	0.1
9	0	0
10	Then stop	Then stop

Citalopram 40mg/ml and escitalopram 20mg/ml liquid are not recommended due to the difficulty with accurately measuring small doses. However, where necessary similar principles could be applied.

© 2024 Kendrick T et al. *JAMA Network Open.*

eReferences 2.

1. Bowers, H., Kendrick, T., van Ginneken, N., et al. A Digital Intervention for Primary Care Practitioners to Support Antidepressant Discontinuation (ADvisor for Health Professionals): Development Study. Journal of Medical Internet Research 2021;23(6):e25537. http://dx.doi.org/10.2196/25537
2. Yardley, L., Morrison, L., Bradbury, K., Muller, I. The Person-Based Approach to Intervention Development: Application to Digital Health-Related Behavior Change Interventions. Journal of Medical Internet Research 2015;17:e30).
3. Michie S, Van Stralen M, West R. The behaviour change wheel: a new method for characterising and designing behaviour change interventions. Implementation Science 2011;6:42 doi:10.1186/1748-5908-6-42.
4. Moore GF, Audrey S, Barker M, Bond L, Bonell C, Hardeman W, Moore L, O'Cathain A, Tinati T, Wight D, Baird J. Process evaluation of complex interventions: Medical Research Council guidance. BMJ 2015;350:h1258. doi: 10.1136/bmj.h1258.

© 2024 Kendrick T et al. *JAMA Network Open.*

eAppendix 3. PWP Telephone Support

Guide for Psychological Practitioners providing telephone support in the REDUCE trial

This guide is intended to help you to ensure that the most important points (in bold) are covered, and to remind you of other useful issues to cover with the patients. You may not have enough time to cover all these issues, so which of these you cover will depend on your assessment of the patient's needs. **It is important that you place a tick in the box next to each point that you do cover during the telephone support**. **Thank you.**

Patients' journey through the trial
The patient Internet intervention (called '*ADvisor*') gives information and advice about antidepressants**. Familiarizing yourself with *ADvisor* will help you to optimize patient engagement with the intervention**. Modules include:

- ✓ Reducing and stopping (intro to the program)
- ✓ How to reduce antidepressants
- ✓ Thinking about antidepressants (their effects and why lifelong treatment may not be necessary)
- ✓ Dealing with withdrawal symptoms
- ✓ Keeping well
- ✓ Thinking about what you value in life
- ✓ Moving forward (see Appendix, last page).

Before call 1: Participating patients will be contacted by the research team, and given advice and support to log on and engage with the web-based support. After looking at the on-line *ADvisor* for patients, they will be asked to arrange to see their GP or nurse practitioner (NP) to discuss coming off antidepressants, including agreeing a time to start tapering the dose, and organizing a first GP/NP follow-up appointment for review. The GP/NP will be aware that the patient has been enrolled into the trial and have a copy of the consent form.

Timing of call 1: The research team will book the patient in for their first support call using the Dr Julian platform for approximately 2 weeks after the initial GP/NP appointment in which they will have agreed a time to start tapering off antidepressant treatment.

Signposting back to the GP/NP: Patients may experience withdrawal symptoms. If the symptoms are interfering with daily activities, they should be advised to speak with their GP/nurse. If the symptoms do not interfere with daily activities, you can advise them to speak to their GP/nurse if the symptoms worsen. The number and timing of subsequent GP/NP consultations during tapering and following drug cessation will be left to the participating GPs/NPs to agree with the patients on an individual basis.

General Practitioner/Nurse Practitioner intervention
The practitioner intervention (called '*ADvisor* Health Professional') is worth familiarizing yourself with prior to the calls. Modules include:

- ✓ Why reduce
- ✓ Broaching the subject
- ✓ When to start tapering
- ✓ Reduction schedules for individual antidepressants
- ✓ Dealing with withdrawal symptoms
- ✓ Dealing with relapse
- ✓ *ADvisor* for patients (a summary)
- ✓ Printable pages on antidepressant reduction regimes
- ✓ Sections of *ADvisor* for patients for recommending to patients.

During the telephone calls, please follow the CARE approach: **C**ongratulate **A**sk **R**eassure **E**ncourage. CARE is simply a shorthand for the supportive, empathic and encouraging general approach we have found works well in our previous research on telephone support.

© 2024 Kendrick T et al. *JAMA Network Open.*

Patient Name:

<u>The first telephone support session.</u>

Up to 30 minutes. To take place at a convenient time between weeks 0 & 2

This telephone contact offers the opportunity to build the patient's self-efficacy and address any barriers.

Date:	Time:	Approximate length of call (mins):	No of attempts:

	Check box
**Call opening**	
1. Ask the patient for consent for the call to be audio recorded.	
2. **Set agenda for the call** (Possible prompts: I'm here to offer support during your tapering, this is the first of three calls, today we will be talking about your tapering, checking in on how you're doing and talking you through *ADvisor* etc).	
3. Ask the patient about the tapering schedule they have discussed with their GP and ask how it is going so far.	
4. Check patient's confidence in and understanding of their tapering schedule (this might emerge in 3. above).	
**PHQ-9**	
5. Introduce PHQ-9 as a questionnaire to gauge how the patient is. Patients may be familiar with the questionnaire from their recruitment into the study. Refer to the email with the questionnaire they have received recently – ask if the patient has thought about their answers. **Check PHQ-9 score and advise seeking help from the GP if the score is above 12.**	
6. If score on **question 9 of the PHQ-9 is 1, 2 or 3** (i.e. not zero), or if **any risks of self-harm, risks to the patient from others, or risks from the patient to others** are identified during the call, **assess risk of suicide/self-harm and follow risk management standard operating procedure**.	
7. Discuss symptoms that score highly for the patient (use it as a vehicle for discussion throughout).	
**Withdrawal and *ADvisor***	
8. Ask if the patient has looked at *ADvisor*, explain how it may be helpful, tailor information to the patient (highlight relevant sections based on withdrawal symptoms/issues the patient has mentioned). Check the patient's understanding and confidence in using *ADvisor* and direct to study team if they need assistance. **Some patients may say they wish to avoid negative information but reinforce that it is there to support and is helpful.**	
9. If they have not arisen in earlier discussion directly ask the patient about any concerns/barriers/resistance to discontinuing. **Respond to expressions about fears of relapse and any withdrawal symptoms**, and reassure where possible, using clinical skills and encouragement, referring the patient to consult the appropriate sections of *ADvisor* where appropriate (see **Appendix** below).	
10. **Remind the patient of the question in *ADvisor* about why they would like to discontinue** (in the section, '*Reducing and Stopping Antidepressants*'). (Possible prompts: "Why might it be good to stop taking antidepressants?"; "How might things improve if you weren't taking antidepressants?"; "What don't you like about taking antidepressants?" etc.).	
**Call closing**	
11. Ask the patient if they have any other concerns/questions about their tapering/*ADvisor*.	
12. **Arrange second telephone contact.** This can be flexible and agreed with the patient. For example, if the patient is not starting to taper immediately, they may prefer to be called when they are starting. This call will be between weeks 2 and 12.	
13. **Who to contact and when:**<br>✓ Tapering, concerns about any symptoms ➔ GP or NP<br>✓ Issues with *ADvisor* /study information ➔	

© 2024 Kendrick T et al. *JAMA Network Open.*

Hannah Bowers, H.M.Bowers@soton.ac.uk, 07XXXXXXXXX<br><br>Riya Tiwari, R.Tiwari@soton.ac.uk, 07XXXXXXXXX<br><br>Amy Din, A.Din@soton.ac.uk, 07XXXXXXXXX<br><br>OR reduce@soton.ac.uk	
14. Confirm that the patient still agrees for the call to be audio recorded.	
15. **Enter details onto the DR Julian platform for the study team's information to briefly update them on the patient's progress.**	

Further information (please use the space below and overleaf to note down the following):
Any issues raised/Difficult questions asked by the patient/Risk management/Any other relevant information

© 2024 Kendrick T et al. *JAMA Network Open.*

Patient Name:

**The second telephone support session**<br><br>*Up to 15 minutes. To take place at a convenient time agreed with the patient.*<br>To check whether patients follow their tapering schedule, support them in the process and encourage to use *ADvisor* to help them to deal with difficulties they may face during withdrawal process.			
**Date:**	**Time:**	**Approximate length of call (mins):**	**No of attempts:**

	Check box
**Call opening**	
1. Ask the patient for consent for the call to be audio recorded.	
2. **Set agenda for the call** (Possible prompts: this is the second of three calls, today we will be talking about your tapering, checking in on how you're doing and talking you through *ADvisor* etc.)	
3. Ask the patient how tapering is going and ensure they are following the schedule. Where necessary, advise the patient to discuss issues with this schedule with their GP.	
4. **Ask the patient about symptoms of depression or anxiety, and any residual withdrawal symptoms:** have they had any? Have they found ways of coping? If they haven't had any, do they feel able to deal with them if they arise?	
**PHQ-9**	
5. Introduce PHQ-9 as a questionnaire to gauge how the patient is doing. Patients may be familiar with the questionnaire from their previous call. Refer to the email with the questionnaire they had received – ask if the patient has thought about their answers.<br>**Check PHQ-9 score and advise seeking help from the GP if the score is above 12.**	
6. If score on **question 9 of the PHQ-9 is 1, 2 or 3** (i.e. not zero), or **if any risks of self-harm, risks to the patient from others, or risks from the patient to others** are identified during the call, **assess risk of suicide/self-harm and follow risk management standard operating procedure**.	
7. Discuss symptoms that score highly for the patient (use it as a vehicle for discussion throughout).	
ADvisor	
8. If the patient stopped tapering/is not following the tapering schedule, explore why this is. Use clinical skills to explore barriers offered by the patient. Encourage them to consult *ADvisor* about reasons to reduce antidepressants if they have concerns.	
9. Ask the patient how they have found managing stressful situations since reducing. Listen, address concerns and reassure. There is information in the Healthy Paths section of *ADvisor* on this. If the patient expresses concerns about depression returning, use clinical skills to discuss relapse prevention (this is also covered in the Keeping Well section of *ADvisor*). **Ask the patient whether they have consulted the '*Keeping Well'* section of *ADvisor* and remind them to complete the sections on warning signs and list of triggers as needed.** Use clinical skills to support patients with this if appropriate.	
**Call closing**	
10. Ask about any other concerns or fears and **respond as appropriate.**	
11. **Arrange third phone contact.** Patient decides when they would like it. This call will be between weeks 2 and 12.	
12. Confirm that the patient still agrees for the call to be audio recorded.	

© 2024 Kendrick T et al. *JAMA Network Open.*

13. **Enter details onto the DR Julian platform for the study team's information to update on the patient's progress.**	

Further information (please use the space below to note down the following):

✓ Any issues raised/Difficult questions asked by the participant/Risk management/Any other relevant information

Patient Name:

**The third telephone support session**<br><br>*Up to 15 minutes. To take place at a convenient time agreed with the patient.*<br>The aim is to build confidence, by ensuring the patient feels that they are capable of managing any future difficulties on their own, or know when to speak to their GP.			
**Date:**	**Time:**	**Approximate length of call (mins):**	**No of attempts:**

	Check box
**Call opening**	
1. Ask the patient for consent for the call to be audio recorded.	
2. **Set agenda for the call** (Possible prompts: This is the third and final call, today we will be checking in on how you're doing and looking ahead to the future etc.).	
3. Ask how the patient's tapering is going. If the patient stopped tapering and is taking the antidepressants again, ask them where they are now in relation to thinking about trying again.	
4. Ask the patient about withdrawal symptoms and use clinical skills to discuss relapse prevention going forward.	
5. At this point many patients will have finished tapering so it might be useful to ask them to reflect on how they feel.<br>✓ If they feel well, highlight that they are feeling well despite no longer taking antidepressants.<br>✓ If they are having any difficulties ensure that they know what they can do to address these (including speaking with the GP if necessary).	
**PHQ-9**	
6. Introduce PHQ-9 as a questionnaire to gauge how the patient is doing. Patients may be familiar with the questionnaire from their previous call. Refer to the email with the questionnaire they had received – ask if the patient has thought about their answers.<br>**Check PHQ-9 score and advise seeking help from the GP if the score is above 12**.	
7. If score on **question 9 of the PHQ-9 is 1, 2 or 3** (i.e. not zero), or **if any risks of self-harm, risks to the patient from others, or risks from the patient to others** are identified during the call, **assess risk of suicide/self-harm and follow risk management standard operating procedure**.	
8. Discuss symptoms that score highly for the patient (use it as a vehicle for discussion throughout).	
ADvisor	
9. Remind patient that *ADvisor* is there as a resource that they can use whenever they would like over the coming months, until the end of the study.	
**Closing**	
10. Ask the patient how they feel about moving forwards after their tapering.	
11. Confirm that the patient still agrees for the call to be audio recorded.	

© 2024 Kendrick T et al. *JAMA Network Open.*

12. Ask the patient if they have any other questions or concerns	
13. **Enter details onto the DR Julian platform for the study team's information to update them on the patient's progress.**	

Further information (please use the space below to note down the following):

✓ Any issues raised/Difficult questions asked by the patient/Risk management/Any other relevant information

© 2024 Kendrick T et al. *JAMA Network Open.*

ADvisor modules for PWP information

ADvisor is an online resource that provides information that patients can click through. This information is divided into 8 sections. These include:

**How ADvisor can help**	• An introduction to the intervention<br>• Explains what support is available in ADvisor and how to use ADvisor
**Reducing and stopping**	• Introduction to reducing and stopping antidepressants<br>• Includes stories of patients who have come off antidepressants<br>• Asks patients to write down their motivations for discontinuing
**Thinking about antidepressants**	• Explains that there are mixed ideas about how antidepressants work<br>• Regardless of how antidepressants work, many people can discontinue without relapsing
**Dealing with withdrawal symptoms**	• An overview of most common symptoms<br>• How to distinguish relapse from withdrawal<br>• Encouraged to 'wait it out' and tolerate mild symptoms<br>• Advised to see GP if they cannot tolerate symptoms
**I'm worried about stopping**	Support for addressing common worries about reducing and stopping:<br>• Fear of relapse<br>• Keeping up with day to day responsibilities<br>• Dealing with life stresses<br>• Needing to restart antidepressants<br>• Worries about previous unsuccessful attempts
**Keeping well**	• Education about how thoughts influence emotions<br>• Guidance on not responding automatically to situations and tolerating difficult feelings using mindfulness techniques<br>• Space to write down their triggers and warning signs for relapse, and a plan for how to respond to prevent relapse
**Thinking about what you value in life**	• Defining values<br>• Help patients identify and write down what they value in life<br>• Guidance and space to write goals in line with what they value in life
**Moving forward**	• Describing what stress is and isn't<br>• 'Paths' that guide the patient to different techniques for managing difficult emotions<br>• Exercises for managing difficult emotions (e.g. mindful walking, scheduling pleasant activities)

© 2024 Kendrick T et al. *JAMA Network Open.*

Analysis of the fidelity of the psychological wellbeing practitioner (PWP) support calls

PWP support calls were provided for 143 (80.3%) of the 178 participants in the intervention arm. In the remaining cases three attempts were made to contact each patient after which it was assumed if they hadn't responded to decline then they did not want the telephone support.

The telephone support calls provided by four PWPs in Work stream 5 (WS5) were checked for fidelity against the guidance provided for 35 calls that took place between 2020 and 2021. Three sets of calls (calls 1, 2 & 3) for each of the PWPs were analysed to determine initial fidelity (two sets of calls each) and any drift from the guide towards the end of the trial (one set of calls each). These calls were audio-recorded, transcribed verbatim, and analysed for fidelity against the guidance provided.

In call 1 (n=12) the PWPs always asked whether the patient had discussed tapering with their GP or NP, how it was going, whether they understood the tapering regime, and whether they had any concerns or questions about the tapering process so far. They always administered the PHQ-9 questionnaire for depressive symptoms. They always asked if the patient had looked at the Advisor online intervention, although this was usually done briefly and not explored in any detail in most calls. All patients agreed to a second call and a date was confirmed. The PWPs scored lower on revisiting consent for the call to be recorded at the end of the call (58% of the time), and on checking with the patient whether they understood who to contact in case of difficulties (50%).

In call 2 (n=12) the PWPs always asked the patient how tapering was going, and whether they had discussed it again with their GP following the initial consultation. They always asked about withdrawal symptoms, and discussed ways of coping with them. They always administered the PHQ-9 a second time. All agreed a date for a third call. They scored lower on asking about, and responding to other patient fears and concerns (83%), and revisiting consent to record on closing the call (25%).

In call 3 (n=11) all patients were again asked about tapering and withdrawal symptoms, and were administered the PHQ-9. They were always asked if they knew who to contact if they had difficulties. They again scored lower on asking about other concerns or fears (82%), and lowest for revisiting consent for the recording at the end of the call (27%).

© 2024 Kendrick T et al. *JAMA Network Open.*
