# Supplementary material for: Internet and Telephone Support for Discontinuing Long-Term Antidepressants: The REDUCE Cluster Randomized Trial
Source: JAMA Netw Open. 2024 Jun 24;7(6):e2418383. doi: 10.1001/jamanetworkopen.2024.18383 (PMC11197448; doi:10.1001/jamanetworkopen.2024.18383)
Supplement: Supplement 3. — Data Sharing Statement [file jamanetwopen-e2418383-s003.pdf]

Data Sharing Statement

Kendrick. Internet and Telephone Support for Discontinuing Long-Term Antidepressants. *JAMA Netw Open*. Published June 24, 2024. doi:10.1001/jamanetworkopen.2024.18383

Data
Data available: Yes
Data types: Deidentified participant data
How to access data: No data will be shared prior to publication of the results of the study in the Journal and in the report to the funder, the NIHR. Applications should be emailed to the Chief Investigator, Tony Kendrick, at ark1@soton.ac.uk
When available: With publication

Supporting Documents
Document types: None

Additional Information
Who can access the data: After publication we will be open to applications for collaborations with other scientists to use the study data, as our research ethics permission is for use of the data by the research team only.
Types of analyses: For any purpose.
Mechanisms of data availability: Applications to use the data will need to be in the form of a peer-reviewed protocol, and will be considered by the three lead investigators, Tony Kendrick at Southampton (ark1@soton.ac.uk), Una Macleod at Hull, and Mark Gabbay at Liverpool, in the first instance, before a decision on data sharing will be taken by the rest of the co-applicants.
Any additional restrictions: Qualitative data will not be shared, due to the relatively high risk of breaches of confidentiality arising from the nature of qualitative interviews, which are difficult to make completely anonymous.
